# Prevalence, characteristics, and clinical course of mpox-related ophthalmic disease (MBOTE-EYE): a prospective cohort study in South Kivu, Democratic Republic of the Congo

**DOI:** 10.1016/j.langlo.2026.104054

**Published:** 2026-08-27

**Authors:** Theophile Kabesha, Christophe Van Dijck, Samuel Mudwanga, Sarah Houben, Yves Mujula, Papy Munganga, Jean-Claude Tshomba, Guy Mukari, Tony Wawina-Bokalanga, Eddy Kinganda-Lusamaki, Bart K Jacobs, Achilleas Tsoumanis, Emmanuel Hasivirwe Vakaniaki, Anne W Rimoin, Isabel Brosius, Elise De Vos, Eugene Bangwen, Stefanie Bracke, Koen Vercauteren, Jean-Claude Mwanza, Deogratias B Ngoma, Patrick Katoto, Steven Yeh, Nelly N Kabedi, Veronique Nussenblatt, Olivier Tshiani-Mbaya, Ian Crozier, Sabin Sabiti Nundu, Jason Kindrachuk, Laurens Liesenborghs, Placide Mbala-Kingebeni

**Affiliations:** Faculty of Medicine, Université Officielle de Bukavu, Bukavu, Democratic Republic of the Congo (T Kabesha PhD, S Mudwanga MD); Department of Clinical Sciences, Institute of Tropical Medicine, Antwerp, Belgium (C Van Dijck PhD, S Houben PhD, T Wawina-Bokalanga PhD, B K Jacobs PhD, A Tsoumanis PhD, E H Vakaniaki MD, I Brosius MD, E De Vos MSc, E Bangwen MSc, S Bracke MD, K Vercauteren PhD, S S Nundu PhD, L Liesenborghs PhD); Data Science Institute, I-BioStat, Hasselt University, Hasselt, Belgium (C Van Dijck PhD); Department of Microbiology, Immunology and Transplantation, KU Leuven, Leuven, Belgium (C Van Dijck PhD, E H Vakaniaki MD, I Brosius MD, E Bangwen MSc, L Liesenborghs PhD); Institut National de Recherche Biomédicale, Kinshasa, Democratic Republic of the Congo (Y Mujula BSc, T Wawina-Bokalanga PhD, E Kinganda-Lusamaki PhD, E H Vakaniaki MD, O Tshiani-Mbaya MD, S S Nundu PhD, Prof P Mbala-Kingebeni PhD); National Police Hospital, Goma, Democratic Republic of the Congo (P Munganga MD); Rodolphe Mérieux Institute, Institut National de Recherche Biomédicale, Goma Laboratory, Goma, Democratic Republic of the Congo (J-C Tshomba BSc); National Program for HIV/AIDS, Ministry of Public Health, Kinshasa, Democratic Republic of the Congo (G Mukari MD); Department of Epidemiology, University of California, Los Angeles, CA, USA (Prof A W Rimoin PhD); Department of Ophthalmology, University of North Carolina, Chapel Hill, NC, USA (Prof J-C Mwanza PhD); Faculty of Medicine, Catholic University of Bukavu, Bukavu, Democratic Republic of the Congo (D B Ngoma MD); Center for Tropical Diseases and Global Health, Faculty of Medicine, Catholic University of Bukavu, Bukavu, Democratic Republic of the Congo (Prof P Katoto PhD); Truhlsen Eye Institute, University of Nebraska Medical Center, Omaha, NE, USA (Prof S Yeh PhD); Department of Ophthalmology, University of Kinshasa, Kinshasa, Democratic Republic of the Congo (N N Kabedi); Laboratory of Clinical Immunology and Microbiology, National Institute of Allergy and Infectious Diseases, Bethesda, MD, USA (V Nussenblatt MD); Clinical Monitoring Research Program Directorate, Frederick National Laboratory for Cancer Research, Frederick, MD, USA (I Crozier MD, O Tshiani-Mbaya MD); National Microbiology Laboratory Branch, Public Health Agency of Canada, Winnipeg, MB, Canada (Prof J Kindrachuk PhD); Service de Microbiologie, Département de Biologie Médicale, Cliniques Universitaires de Kinshasa, Université de Kinshasa, Kinshasa, Democratic Republic of the Congo (E Kinganda-Lusamaki PhD); University of Kinshasa Medical School, Kinshasa, Democratic Republic of the Congo (Prof P Mbala-Kingebeni PhD); TransVIHMI, Université de Montpellier, INSERM, IRD, Montpellier, France (E Kinganda-Lusamaki PhD); Department of Medical Microbiology & Infectious Diseases, University of Manitoba, Winnipeg, MB, Canada (Prof J Kindrachuk PhD); South African National Bioinformatics Institute, University of the Western Cape, Cape Town, South Africa (Prof P Mbala-Kingebeni PhD); Department of Virology, Graduate School of Medicine, Osaka Metropolitan University, Osaka, Japan (Prof P Mbala-Kingebeni PhD)

## Abstract

**Background:**

Systematic longitudinal ophthalmologist-led assessment of mpox-related ophthalmic disease (MPOXROD) is rarely reported. We aimed to characterise the prevalence and clinical course of MPOXROD under supportive management during a clade Ib outbreak.

**Methods:**

MBOTE-EYE was a substudy nested within a prospective cohort in South Kivu, Democratic Republic of the Congo. All hospitalised patients with PCR-confirmed mpox (within the last 48 h) were eligible. Participants underwent ophthalmological examination at enrolment, discharge, and days 29 and 59. Conjunctival swabs were collected for monkeypox virus PCR. Risk factors for MPOXROD were assessed using mixed-effects Poisson regression.

**Findings:**

Between Oct 28, 2024, and June 30, 2025, 310 participants were enrolled (median age 14·0 years [IQR 2·5–25·0]; 166 [54%] female). At enrolment, conjunctival disease was present in 112 (36% [95% CI 31–42]) participants, corneal disease in 24 (8% [5–11]), and anterior uveitis in two (1% [0–2]). Overall, 134 (43% [38–49]) participants developed MPOXROD during follow-up, most often bilaterally. Blindness following ulcerative keratitis occurred in two (1% [0–6]) of 225 participants. Periorbital lesions (adjusted risk ratio [aRR] 3·65 [2·42–5·49]) and severe acute malnutrition (aRR 3·16 [1·22–8·22]) were associated with MPOXROD. PCR was done among 89 patients and cycle threshold values below 25 occurred exclusively during active MPOXROD.

**Interpretation:**

Ophthalmic involvement in clade Ib mpox is common and frequently bilateral, with a substantial burden of keratitis and risk of vision loss. Systematic eye examinations should be integrated into mpox care, and targeted ophthalmic therapies should be developed.

## Introduction

Mpox, caused by monkeypox virus (MPXV) infection, is characterised by fever, skin rash, and lymphadenopathy.^1,2^ The clinical manifestations of mpox can resemble milder forms of smallpox, which was caused by the now eradicated variola virus, another member of the Orthopoxvirus genus that also includes MPXV.^1,2^ Since 2017, several large outbreaks of mpox have occurred with extensive human-to-human transmission, mainly driven by sexual contact, and with international spread.^1,3,4^ Between 2017 and 2026, WHO declared two mpox-related public health emergencies of international concern.^5^ Clades Ia, Ib, and IIb MPXV continue to circulate among dense populations, including sexual networks with over 50 000 cases in 2025 alone.^6^

Although mpox is associated with generally low case fatality and resolution of clinical symptoms and signs within 2–3 weeks, complications such as bacterial superinfection, large necrotising skin lesions, dehydration, or proctitis can cause severe disability or death in a small minority of cases, with a higher risk in immunocompromised individuals.^2,7^ Mpox has also been associated with poor pregnancy outcomes.^8^ In addition, mpox-related ophthalmic disease (MPOXROD) has been described to cause significant morbidity. Studies have reported clade I mpox-related conjunctivitis in about 22% of patients, and keratitis in about 3%, with approximately 4% of patients developing visual impairment due to corneal scarring or phthisis bulbi.^9^ In the absence of effective antiviral treatments, management of MPOXROD is limited to supportive care that includes topical lubrication and anti-inflammatory or antibiotic therapies.^10^

To date, and to the best of our knowledge, systematic longitudinal characterisation of clade I MPXV-associated ophthalmic manifestations based on expert ophthalmological evaluation is not available. Therefore, we aimed to prospectively characterise MPOXROD prevalence, clinical and virological features, and clinical course under supportive management in a cohort of patients infected with clade I MPXV in South Kivu, Democratic Republic of the Congo.

## Methods

### Study design and participants

This study was implemented in Kamituga, South Kivu, Democratic Republic of the Congo, during a clade Ib mpox epidemic that has been ongoing since September, 2023. For reasons of isolation, all patients with PCR-confirmed mpox were hospitalised in a dedicated mpox treatment centre at Kamituga General Hospital. Consenting individuals were enrolled in two parallel observational studies. The MBOTE-Kamituga study (NCT06652646) is an ongoing prospective clinical characterisation study of individuals with suspected MPXV infection initiated on May 2, 2024, with interim results reported previously.^7^ In addition, from Oct 28, 2024 onwards, MBOTE-Kamituga participants of any age or sex (self-reported [male or female]) with confirmed mpox, defined as a positive MPXV PCR on a sample collected in the last 48 h, could be enrolled in the ophthalmological substudy, MBOTE-EYE (NCT06579885). The primary objective of MBOTE-EYE was to determine the prevalence of MPOXROD at presentation. Secondary objectives were to describe ophthalmic symptoms and signs, visual acuity, and the presence of MPXV DNA in conjunctival swabs at presentation and during follow-up.

MBOTE-Kamituga and MBOTE-EYE were approved by the Ethics Committee of the University of Kinshasa (approval IDs ESP/CE/78/2024 and ESP/CE/083/2024, respectively) and the University Hospital Antwerp (IDs 6383 and 6440, respectively). MBOTE-EYE was additionally approved by the University of Manitoba Research Ethics Board (ID HS26717). Before all study procedures, written consent was obtained from participants aged 18 years and older, and from parents or guardians for minors. Children aged 12–17 years received age-appropriate information and signed an assent form. Specific consent was also obtained for photographs presented for publication. All participants received reimbursement of transportation costs, and free care and nutrition, including topical ophthalmological therapies.

### Procedures

Participants were evaluated by an MBOTE-Kamituga study physician, who collected sociodemographic and medical history data, as well as symptoms and signs, including the WHO severity score.^11^ Next, they were examined by a dedicated MBOTE-EYE study ophthalmologist, who collected data on ophthalmic symptoms and signs, including evaluation of visual acuity (optotype test, from age 3 years onwards, if understanding of the child allowed); pupillary light reflexes and ocular motility; intraocular pressure (TonoPen tonometer [Reichert Technologies, Depew, NY, USA]); portable slit lamp examination of the anterior segment before and after application of fluorescein; and dilated indirect fundoscopic examination and photographic imaging of the eyes and orbits using iPhone 13. No data on race or ethnicity were collected.

All data were prospectively captured using standardised case report forms in REDCap (Vanderbilt University, Nashville, TN, USA). Between Dec 19, 2024, and Jan 10, 2025, MBOTE-Kamituga was paused while MBOTE-EYE continued, resulting in missing data on the medical histories of 48 participants.

The study ophthalmologist also collected conjunctival swabs of the surface of the lower conjunctival sac from all participants at all visits using minitip FLOQSwabs (Copan, Brescia, Italy). Swabs were immediately placed into 1 mL of universal transport medium and samples were stored at −20°C. Regional political instability limited the shipment of materials to and from the study area, necessitating the discontinuation of conjunctival swab collection from Dec 5, 2024 onwards.

Conjunctival swabs were shipped to the Institut National de Recherche Biomédicale, Kinshasa, for PCR analysis using the RADI Fast MPXV Detection Kit (RV015R, KH Medical, Seoul, South Korea). Cycle threshold (Ct) values were used as semi-quantitative estimates of viral concentrations (higher values corresponding to lower concentrations). Ct values of 40 and above were considered PCR-negative.

HIV testing was performed at the discretion of the treating physician, and ophthalmic treatments, including topical corticosteroids, were prescribed at the discretion of the treating ophthalmologist based on clinical assessment. Patients were discharged at the discretion of the treating physician. Participants were invited for outpatient follow-up visits at days 29 and 59 post-diagnosis. In case of new or worsening ocular symptoms (reduced visual acuity, photophobia, eye pain, or eyelid lesion or oedema) between enrolment and day 59, an additional study visit was performed.

### Statistical analysis

The minimum required sample size was estimated at 246 participants, assuming an MPOXROD prevalence of 20%, a 95% confidence level, and a 5% precision.

Demographic, clinical, virological, and ophthalmological characteristics were summarised using medians with IQR for continuous variables and proportions for categorical variables. Baseline characteristics were presented by age group: children younger than 5 years, children aged 5–14 years, and individuals older than 14 years. Descriptive analyses were conducted at the participant level, classifying ophthalmic conditions as present if observed in either eye. MPOXROD was defined as the presence of conjunctival disease (ie, conjunctivitis or conjunctival mpox lesions), scleritis, corneal disease (ie, keratitis, corneal epithelial defects, oedema, or opacities), uveitis, or optic nerve involvement.

For post-hoc exploratory analyses, we evaluated risk factors for MPOXROD, and for reduced visual acuity. Analyses were performed at the eye-timepoint level, including repeated observations for each eye across study visits. Associations between risk factors and MPOXROD were assessed using generalised linear mixed-effects models with a Poisson distribution, specifying MPOXROD as a binary outcome. Random intercepts were included for eye (left or right) nested within participant ID to account for within-participant and within-eye correlation. Risk factors included age group; sex; malnutrition status; presence of mpox lesions on the periorbita, palpebra, or hands; WHO severity score (total body lesion count <25 [mild], 25–99 [moderate], 100–250 [severe], or >250 [grave]); and time-varying covariates (conjunctival swab PCR-positivity, Ct value, and time since symptom onset), which were included using their values at the corresponding visit. Each risk factor was evaluated in a separate multivariable model. For each model, adjustment variables were prespecified a priori based on causal assumptions informed by directed acyclic graphs (appendix 4 p 3), and included age, sex, malnutrition status, and time since symptom onset. Results were presented as adjusted risk ratios (aRR) with 95% CIs, calculated using robust standard errors. In a secondary analysis, we used a similar model with reduced visual acuity (optotype score <8/10) as the outcome, and MPOXROD as the predictor. Results of this model were presented as risk ratios (RR) with 95% CIs, calculated using robust standard errors.

Apart from the regression modelling, which relied on a complete-case approach, all other analyses were done on an available-case basis, with denominators representing the number of individuals with available data for that variable. Statistical analyses were performed using R software (version 4.4.2).

### Role of the funding source

The study funders had no role in the study design, data collection, data analysis, data interpretation, writing of the report or the decision to submit the report.

## Results

Between Oct 28, 2024, and June 30, 2025, 320 individuals were screened for eligibility, and 310 (97%) were enrolled in MBOTE-EYE (figure 1). About half (166 [54%]) were female, and the median age was 14·0 years (IQR 2·5–25·0). Comorbidities included acute malnutrition (n=22), HIV infection (n=3), renal disease (n=2), tuberculosis (n=2), hepatic disease (n=1), and diabetes (n=1; table 1). 12 (5%) of 262 participants received a vaccinia-based vaccine before the development of mpox symptoms (n=3 smallpox vaccination >40 years earlier; n=9 modified vaccinia Ankara–Bavarian Nordic vaccination in 2025). 37 (12%) of 310 participants reported one or more pre-existing ophthalmic conditions at enrolment, including refractive error (n=22), pterygium (n=8), cataract (n=5), glaucoma (n=2), conjunctival melanoma (n=1), and pinguecula (n=1).

Enrolment occurred at a median of 6·0 days (IQR 4·0–8·0) following mpox symptom onset. Mpox severity was mild to moderate (<100 lesions) in 155 (59%) of 262 cases. At enrolment, the most common ophthalmic symptoms were itchiness (64 [21%] of 310), photophobia (62 [20%]), redness (39 [13%]), pain (36 [12%]), decreased visual acuity (24 [8%]), and tearing (17 [6%]; appendix 4 pp 5–6). Other symptoms occurred in less than 5% of participants.

Among participants at enrolment, 78 (25%) of 310 had periorbital and 60 (19%) had palpebral mpox lesions (figure 2A). Conjunctival disease was present in 112 (36% [95% CI 31–42]), corneal disease in 24 (8% [5–11]), and anterior uveitis in two (1% [0–2]; table 2, figure 2B–E, appendix 4 pp 4, 7). Two (1% [0–3]) participants had optic nerve pallor associated with pre-existing glaucoma that was considered unrelated to mpox. No other participants had posterior segment abnormalities.

During the study, participants received topical treatments, including antibiotics (96 [31%] of 310), sodium cromoglycate (57 [18%]), non-steroidal anti-inflammatory drugs (52 [17%]), artificial tears (28 [9%]), and prednisolone (24 [8%]; appendix 4 p 8). No antivirals were available. Hospital admission had a median duration of 11 days (IQR 8–16). Ocular symptoms and signs usually improved during hospitalisation, except among 18 participants who were re-evaluated because of new or increasing ocular pain (n=10), palpebral oedema or ocular mpox lesions (n=9), reduced visual acuity (n=4), or photophobia (n=2), with some participants experiencing more than one indication. During such unscheduled visits, eight additional patients were diagnosed with conjunctival disease, five with palpebral mpox lesions, and two with corneal disease. Four patients (all younger than 5 years) died before hospital discharge and two left the hospital at their own initiative. The remaining 304 (98%) were examined at hospital discharge. Among participants with conjunctival, corneal, and uveal involvement during hospitalisation, residual disease at discharge was observed in 20 (17%) of 117, nine (36%) of 25, and two (100%) of two, respectively.

Among participants attending outpatient follow-up, conjunctival disease was observed in 26 (10% [95% CI 7–15]) and corneal disease in eight (3% [2–6]) of 254 at day 29, decreasing to 14 (6% [4–10]) and four (2% [1–5]) of 224 at day 59, respectively. Anterior uveitis persisted in one participant at both timepoints (0% [0–2] at day 29 and 0% [0–3] at day 59). The second participant diagnosed with anterior uveitis was lost to follow-up after hospital discharge.

Overall, during the observation period, 134 (43% [95% CI 38–49]) of 310 participants developed MPOXROD, of whom five (4%) and 129 (96%) had unilateral and bilateral disease, respectively. Signs of MPOXROD were often already present at enrolment (215 [82%] of 263 eyes). Within the limits of our observation period, although corneal disease frequently co-occurred with conjunctival disease at some stage (37 [79%] of 47 eyes), it was rarely preceded by a phase of milder eye disease such as conjunctival disease (eight [17%] of 47 eyes). Among the 218 participants for whom optotype charts could be used, visual acuity scored below 8/10 at least once during follow-up in 50 (23% [95% CI 18–29]) and below 3/10 in seven (3% [1–7]). Two of 224 individuals (1% [0–6]) had persistent blindness (visual acuity score <1/20) at day 59 following ulcerative keratitis. Active MPOXROD was a significant risk factor for a visual acuity score below 8/10 (RR 1·20, 95% CI 1·03–1·41; figure 3).

MPXV PCR results were available from 312 conjunctival swabs of 89 participants collected throughout study follow-up until swab collection was discontinued: at enrolment, 132 (74%) of 178 swabs from 89 participants were PCR-positive with median PCR Ct values of 31·7 (IQR 28·9–34·7); at discharge, 65 (63%) of 104 swabs from 53 participants were PCR-positive with median Ct values of 33·8 (32·7–35·3); and at day 29, nine (30%) of 30 swabs from 15 participants were PCR-positive with median Ct values of 35·0 (34·7–36·4; figure 4, appendix 4 p 9). No swabs were collected at day 59. PCR Ct values below 25 were observed exclusively among individuals with active signs of MPOXROD.

In adjusted analyses, children younger than 5 years had a higher risk of MPOXROD compared with those older than 14 years (aRR 1·95 [95% CI 1·28–2·98]), whereas those aged 5–14 years had a comparable risk (aRR 0·98 [0·58–1·64]; appendix 4 p 10). The presence of palpebral and periorbital mpox lesions was associated with MPOXROD (aRR 2·10 [1·50–2·94] and 3·65 [2·42–5·49], respectively), whereas lesions on the hands were not (aRR 0·81 [0·54–1·21]). Severe acute malnutrition was also associated with an increased risk of MPOXROD (aRR 3·16 [1·22–8·22]). There was no evidence of an association with sex (aRR for males *vs* females 1·09 [0 ·75–1·59]). There was evidence of interaction between conjunctival PCR positivity and Ct value. Among PCR-positive eyes, higher inverted Ct values (reflecting higher viral load) were associated with an increased risk of MPOXROD (aRR 1·12 [1·04–1·19] per unit increase). Total body lesion count (WHO severity score) was not associated with MPOXROD.

## Discussion

To our knowledge, this is the first study to prospectively conduct systematic longitudinal ophthalmological assessments during an ongoing clade Ib mpox outbreak. Ocular pathology was common, affecting nearly half of individuals with MPXV infection. Conjunctival disease was the most common manifestation. However, approximately one in ten participants presented with severe ophthalmological disease, including ulcerative keratitis and, more rarely, uveitis. The prevalence of specific manifestations of MPOXROD in our study exceeds those reported in a systematic review of 36 observational studies of mpox published between 1980 and 2025, which reported pooled prevalences of 21·9% (95% CI 14·7–29·9) for clade I mpox-related conjunctivitis and of 2·9% (1·4–4·8) for keratitis.^9^ These lower prevalence estimates likely reflect under-ascertainment in the absence of systematic dedicated ophthalmological assessment in earlier studies or differences in outbreak characteristics, health-care-seeking behaviour, and patient population. The prevalences observed in our study are more consistent with findings from another study conducted during the same clade Ib MPXV outbreak in South Kivu, in which individuals underwent ophthalmological evaluation at the time of diagnosis. In that study, conjunctivitis was reported in approximately 30% of cases, and keratitis in nearly 5%.^12^ Overall, consistent with previous descriptions of MPOXROD, ocular involvement was largely confined to the anterior segment, particularly the ocular surface,^9^ and we did not observe rare manifestations described in variola or vaccinia virus infections, including retinitis, chorioretinitis, optic neuropathy, or ophthalmoplegia.^13^

Although severe mpox-related keratitis leading to vision loss has been documented in both immunocompetent and immunocompromised individuals,^9^ the risk factors for MPOXROD remain poorly understood. In our study, children younger than 5 years had approximately double the risk of MPOXROD compared with those older than 14 years, which is in line with the higher incidence of haemodynamic, pulmonary, and neurological complications observed in this age group.^7^ Additionally, severe acute malnutrition was associated with MPOXROD, suggesting that host factors influencing barrier integrity or immune responses might modify susceptibility to or severity of ocular involvement.

Although the pathophysiology of MPOXROD is incompletely defined, several non-mutually exclusive mechanisms are plausible. First, ocular involvement might result from exogenous inoculation of MPXV onto the ocular surface through infectious secretions, hands, or fomites. This mechanism is biologically plausible given the mucosal tropism of orthopoxviruses and the relative immunological vulnerability of the conjunctiva. The higher risk of MPOXROD among individuals with periorbital or palpebral lesions and the predominance of ocular surface disease support this hypothesis, suggesting local autoinoculation or contiguous spread.

Second, MPOXROD might arise endogenously following hematogenous viral dissemination, or through secondary immune-mediated inflammation affecting ocular tissues in the context of systemic immune activation. However, the absence of posterior intraocular disease (eg, retinitis or vitritis) and the overall low prevalence of uveitis do not support these mechanisms as predominant contributors to pathogenesis. The predominantly bilateral presentation noted in our study could be compatible with both exogenous and endogenous routes.

Once ocular involvement is established, evidence from our study and from published case reports of persistent and severe MPOXROD are consistent with a possible role for viral replication in pathogenesis. The immune-privileged nature of the ocular surface might contribute to this viral replication, although its implications remain uncertain.^14,15^ In our study, low PCR Ct values were observed exclusively in individuals with active MPOXROD, particularly with corneal involvement. Case reports of individuals with clade IIb MPXV infection have documented the persistence of MPXV DNA in ocular surface specimens for several months,^16–24^ with replication-competent virus cultured from corneal scrapings up to 145 days after symptom onset.^18,24^ Notably, histopathological analyses of severe mpox conjunctivitis have demonstrated necro-ulcerative inflammation with abundant intralesional orthopoxviral antigen.^25^ In contrast, several participants in our study with ophthalmic manifestations had negative conjunctival PCR results. This might reflect sampling limitations, deeper tissue involvement, or immune-mediated processes, rather than active surface replication. Conversely, detectable MPXV DNA in individuals without ocular disease likely represents low-level contamination rather than true infection. Overall, conjunctival PCR positivity appears neither sensitive nor specific for MPOXROD, although higher viral loads might be a risk factor for clinically apparent disease.

There are currently no antivirals proven effective for the treatment of mpox, and none have been evaluated for MPOXROD in controlled clinical trials. Tecovirimat did not shorten the time to lesion resolution in two clinical trials (PALM007 and STOMP).^26,27^ Nevertheless, given their in-vitro potency and in-vivo activity in animal models, topical high-dose antivirals including tecovirimat, trifluridine, and (brin)cidofovir merit further evaluation.^28^ However, powering randomised trials will be challenging: most MPOXROD is mild and self-limiting, and severe keratitis—the form most likely to benefit from antiviral therapy—is relatively uncommon. Preventive therapy is also unlikely to succeed as ocular involvement including keratitis is typically evident at first presentation. Large numbers of cases with keratitis (n>100) would be required to detect a clinically meaningful benefit in a therapeutic study, as demonstrated in trials for herpetic eye disease.^29,30^

Limitations of our study include the limited capacity to evaluate alternative causes of acute ophthalmic disease, which might have led to an overestimation of the prevalence of MPOXROD, as well as the limited capacity to assess underlying immunosuppression, including HIV infection. In addition, challenges related to sample collection, storage, and transport in a remote setting might have affected data quality; in particular, temperature fluctuations during storage and transport could have contributed to variability in PCR results. Due to the occurrence of an active conflict in the region during the study, supply chains were compromised and conjunctival swabs were obtained from only the first 89 participants, which restricted the sample size for virological analyses and, by definition, constrained the analysis. Furthermore, as viral culture could not be performed, we could not determine whether the PCR-detected viral DNA reflected the presence of infectious virus. Any inferences regarding viral dynamics and the pathogenesis of MPOXROD should therefore be interpreted with caution. Our study included only hospitalised patients with clade Ib MPXV infection in a single health zone in the Democratic Republic of the Congo; caution is advised in generalising our results to patients elsewhere, or patients with clade Ia or clade II MPXV infections given the recognised differences in disease severity and clinical phenotypes. Key strengths of our study include the prospective and systematic ophthalmological assessment of all confirmed mpox cases, with longitudinal clinical and virological follow-up throughout the full clinical course, providing an unprecedented dataset to inform the prevalence, risk factors, and clinical course of MPOXROD under supportive management.

Ophthalmic disease in individuals affected by mpox remains a clinical challenge and appears to disproportionately affect young children and individuals with severe acute malnutrition. Our study elucidates key aspects that might offer insights into the pathophysiology and clinical course of MPOXROD under supportive management that could inform the design of protocols for evaluating ophthalmic therapeutic approaches, including candidate topical antivirals.

## Supplementary Material

3

5

2

1

4

## Figures and Tables

**Figure 1:**
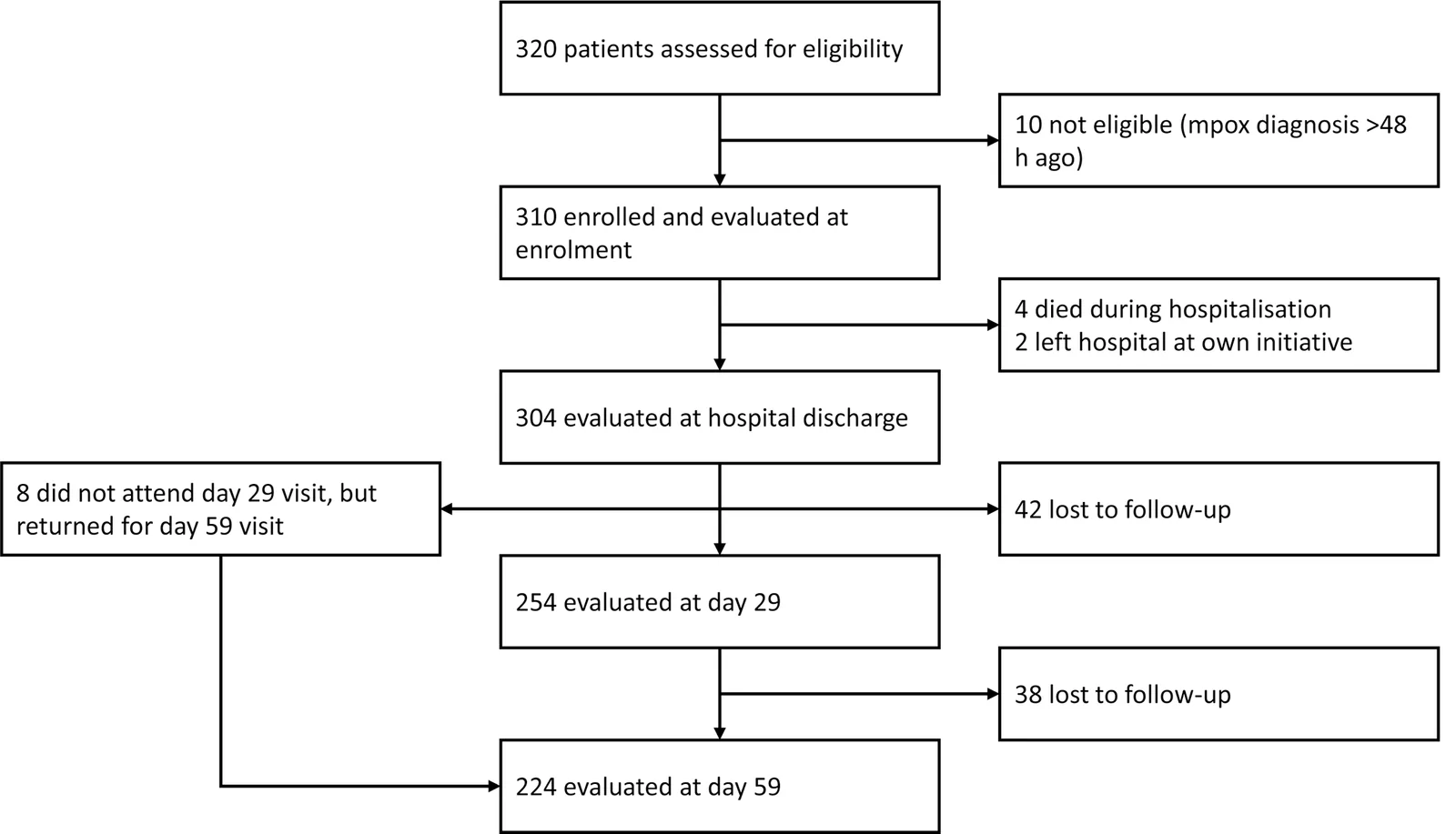
Participant enrolment and follow-up

**Figure 2:**
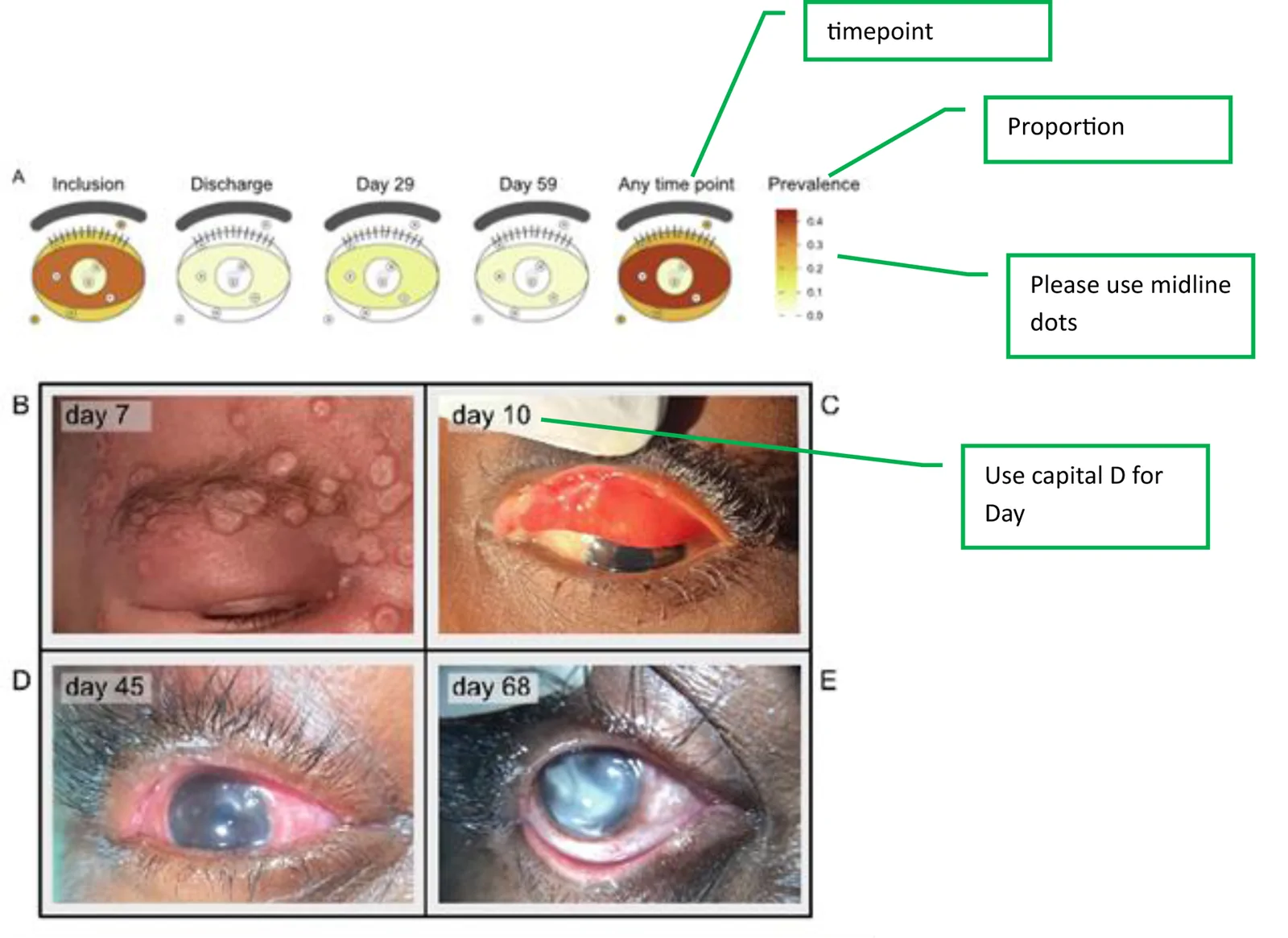
Mpox-related ophthalmic disease features (A) Anatomical heatmap showing prevalence of signs of inflammation (surface fill) or mpox lesions or ulcers (small circles) on the cornea, conjunctiva, palpebra, and periorbita in all study participants over time. (B–E) Ophthalmic complications in different individuals. (B) Right eye: extensive periorbital and palpebral mpox lesions with blepharitis. (C) Left eye: mpox lesion on the palpebral mucosa of the upper eyelid. (D) Right eye: ulcerative keratitis. (E) Right eye: post-keratitis corneal opacification (scarring). [Prod: this file shows the layout of figure 2: • For 2A, you should already have SVG versions of the eye diagrams (as they were part of the original submission files), and I have also attached PDF versions. Neither the SVG nor PDF versions seem to be editable (authors have been unable to provide editable versions), but the PDF versions seem better because they contain an editable key, and also they lack the digital artefact that the SVG files have (two horizontal white lines running across each diagram) • For 2B–E, you should already have an EPS version, but I have also attached an additional EPS version with no labels, as well as the individual photographs as TIFF files in case needed (I cropped them to match the images above)

**Figure 3:**
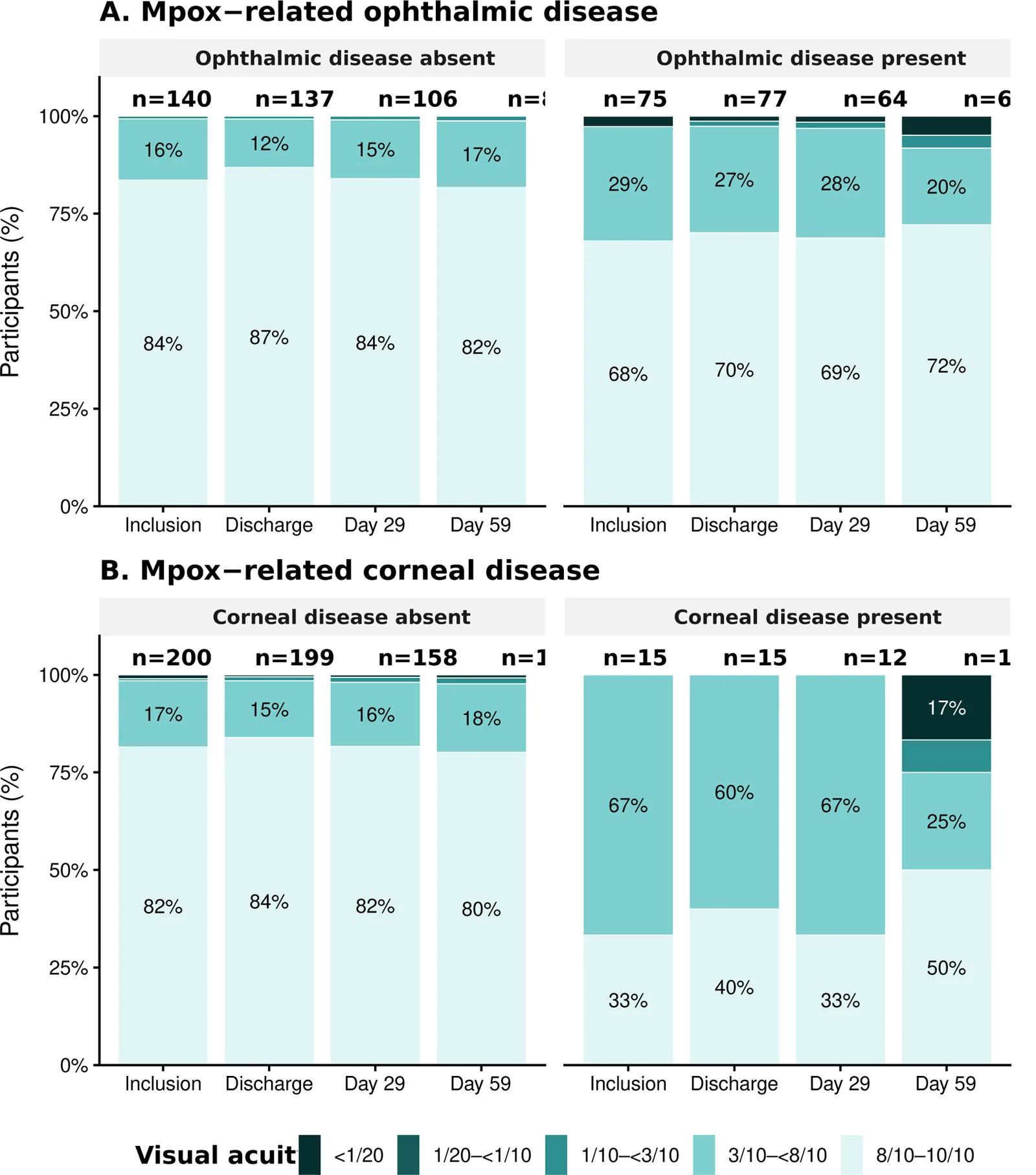
Visual acuity over time among individuals who could be evaluated using an optotype chart, stratified by mpox-related ophthalmic disease (A), and mpox-related corneal disease (B)

**Figure 4:**
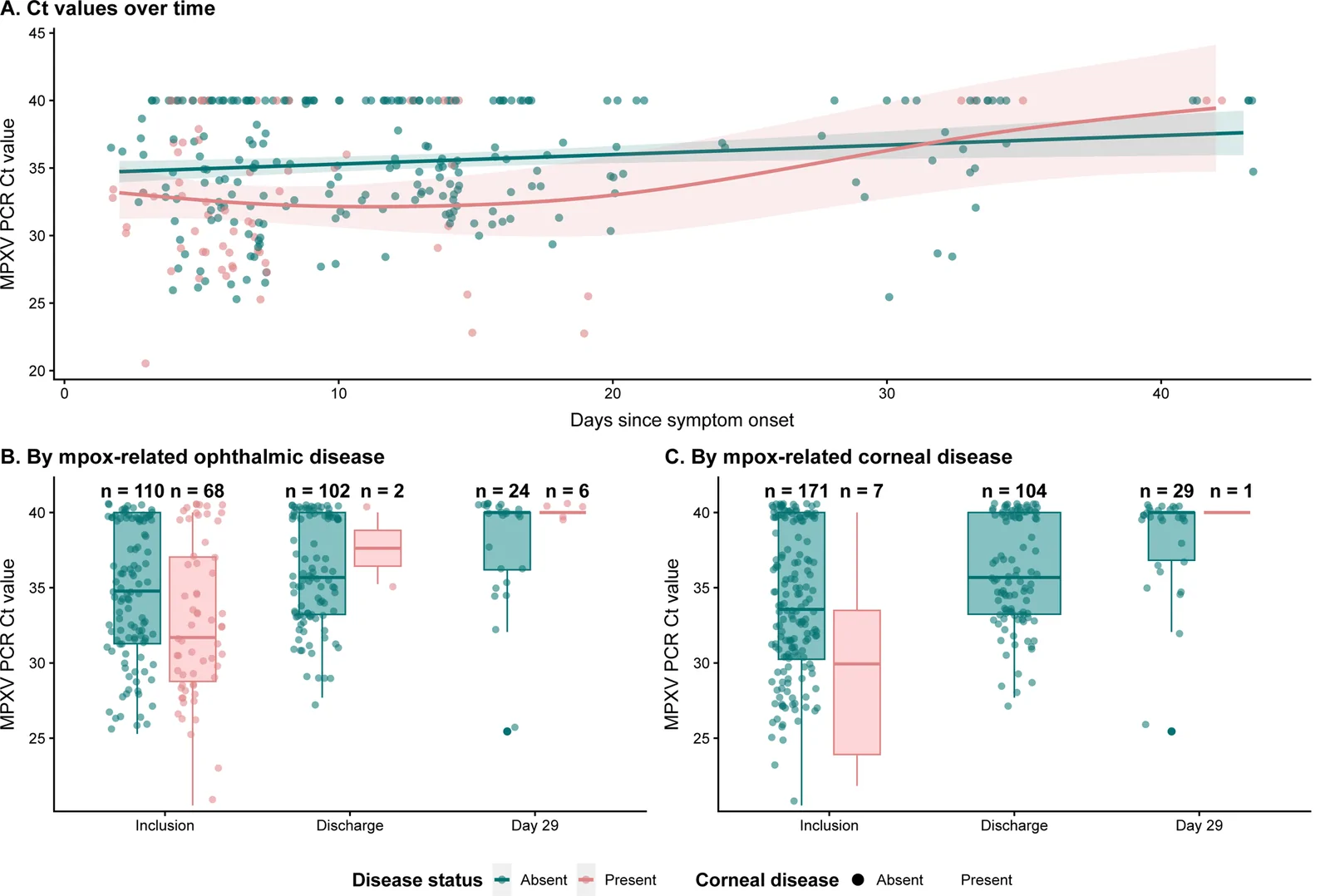
Conjunctival MPXV PCR Ct values over time, stratified by ophthalmic manifestations (A) Individual Ct values plotted against days since symptom onset, with generalised additive model smooths shown by mpox-related ophthalmic disease status. (B) Distribution of Ct values at scheduled visits according to mpox-related ophthalmic disease status. (C) Distribution of Ct values at scheduled visits according to mpox-related corneal disease status. Ct=cycle threshold. MPXV=monkeypox virus.

**Table 1:** Baseline participant characteristics

	Age <5 years (n=105)	Age 5–14 years (n=56)	Age >14 years (n=149)	Overall (n=310)
Median age, years (IQR)	1·2 (0·6–2·5)	8·0 (6·8–12·0)	25·0 (21·0–30·0)	14·0 (2·5–25·0)
Sex				
Female	51/105 (49%)	31/56 (55%)	84/149 (56%)	166/310 (54%)
Male	54/105 (51%)	25/56 (45%)	65/149 (44%)	144/310 (46%)
Missing record in MBOTE-Kamituga study*	17	7	24	48
Median time since onset of symptoms, days (IQR)	6·5 (4·0–8·0)	5·0 (4·0–7·0)	7·0 (4·0–9·0)	6·0 (4·0–8·0)
Median time since onset of skin lesions, days (IQR)	4·5 (3·0–6·0)	4·0 (3·0–5·0)	5·0 (3·0–7·0)	4·0 (3·0–7·0)
Comorbidities				
HIV infection	0/88	1/49 (2%)	2/125 (2%)	3/262 (1%)
Diabetes	0/88	0/49	1/125 (1%)	1/262 (0%)
Tuberculosis	0/8	0/49	2/125 (2%)	2/262 (1%)
Hepatic disease	0/88	1/49 (2%)	0/125	1/262 (0%)
Renal disease	0/88	1/49 (2%)	1/125 (1%)	2/262 (1%)
Acute malnutrition^†^				
Moderate	4/86 (5%)	1/49 (2%)	10/123 (8%)	15/258 (6%)
Severe	2/86 (2%)	3/49 (6%)	2/123 (2%)	7/258 (3%)
Vaccination status				
Received smallpox vaccination during childhood	0/88	0/49	3/117 (3%)	3/254 (1%)
Received MVA-BN vaccination	0/88	0/49	9/125 (7%)	9/262 (3%)
Mpox severity (total number of lesions)				
Mild (<25 lesions)	22/88 (25%)	11/49 (22%)	45/125 (36%)	78/262 (30%)
Moderate (25–99 lesions)	26/88 (30%)	15/49 (31%)	36/125 (29%)	77/262 (29%)
Severe (100–250 lesions)	19/88 (22%)	14/49 (29%)	25/125 (20%)	58/262 (22%)
Grave (>250 lesions)	21/88 (24%)	9/49 (18%)	19/125 (15%)	49/262 (19%)
Previous ophthalmological history in at least one eye				
None	104/105 (99%)	54/56 (96%)	116/149 (78%)	273/310 (88%)
Refractive error	1/105 (1%)	2/56 (4%)	19/149 (13%)	22/310 (17%)
Pterygium	0/105	0/56	8/149 (5%)	8/310 (3%)
Cataract	0/105	0/56	5/149 (3%)	5/310 (2%)
Dry eyes	0/105	0/56	4/149 (3%)	4/310 (1%)
Glaucoma	0/105	0/56	2/149 (1%)	2/310 (1%)
Melanoma	0/105	0/56	1/149 (1%)	1/310 (0%)
Pinguecula	0/105	0/56	1/149 (1%)	1/310 (0%)
Used topical ophthalmological therapy <24 h before enrolment				
Corticosteroids	0/105	1/56 (2%)	1/149 (1%)	2/310 (1%)
Antibiotics (tetracycline)	4/105 (4%)	0/56	0/149	4/310 (1%)
Traditional medicines	0/105	0/56	1/149 (1%)	1/310 (0%)

Data are n/N (%) unless otherwise specified. MVA-BN=modified vaccinia Ankara–Bavarian Nordic.

* Due to pause from Dec 19, 2024, to Jan 10, 2025.

† Moderate acute malnutrition was defined as a mid-upper arm circumference (MUAC) of 11·5–12·5 cm in children younger than 5 years; BMI-for-age Z score −3 to −2 SD in participants aged 5–19 years; and BMI 16–18 kg/m^2^ in adults older than 19 years. Severe acute malnutrition was defined as MUAC less than 11·5 cm in children younger than 5 years; BMI-for-age Z score less than −3 SD in participants aged 5–19 years; and BMI less than 16 kg/m^2^ in adults older than 19 years.

**Table 2:** Ophthalmic outcomes, per participant

	Inclusion	Discharge	Day 29	Day 59	Any timepoint*
Median time since onset of symptoms, days (IQR)	6·0 (4·0–8·0)	16·0 (13·0–22·0)	34·0 (32·0–36·0)	64·0 (62·0–66·0)	NA
Involvement of periorbita and palpebra					
Periorbital mpox lesions	78/310 (25% [21–30])	0/304 (0% [0–1])	0/254 (0% [0–2])	0/224 (0% [0–2])	79/310 (26% [21–31])
Blepharitis	20/310 (7% [4–10])	4/304 (1% [1–3])	3/254 (1% [0–3])	3/224 (1% [1–4])	20/310 (7% [4–10])
Palpebral mpox lesions	60/310 (19% [15 –24])	1/304 (0% [0–2])	0/254 (0% [0–2])	1/224 (0% [0–3])	66/310 (21% [17–26])
Mpox-related ophthalmic disease	118/310 (38% [33–44])	23/304 (8% [5–11])	29/254 (11% [8–16])	16/224 (7% [4–11])	134/310 (43% [38–49])
Conjunctival disease	112/310 (36% [31–42])	20/304 (7% [4–10])	26/254 (10% [7–15])	14/224 (6% [4–10])	132/310 (43% [37–48])
Conjunctivitis without conjunctival mpox lesions	102/112 (91% [84–95])	16/20 (80% [58–92])	23/26 (89% [71–96])	13/14 (93% [69–100])	119/132 (90% [84–94])
Conjunctival mpox lesion(s)with or without conjunctivitis	10/112 (9% [4·9–16])	4/20 (20% [8–42])	3/26 (12% [4–29])	1/14 (7% [0–32])	13/132 (10% [6–16])
Scleritis	0/310 (0% [0–1])	0/304 (0% [0–1])	0/254 (0% [0–2])	0/224 (0% [0–2])	0/310 (0% [0–1])
Corneal disease	24/310 (8% [5 –11])	9/304 (3% [2–6])	8/254 (3% [2–6])	4/224 (2% [1–5])	29/310 (9% [7–13])
Without epithelial defect	2/24 (8% [2–26])	0/9 (0% [0–30])	0/8 (0% [0–32])	2/4 (50% [15–85])	2/29 (7% [2–22])
With epithelial defect	22/24 (92% [74–98])	9/9 (100% [70–100])	8/8 (100% [68–100])	2/4 (50% [15–85])	27/29 (93% [78–98])
Anterior uveitis	2/310 (1% [0–2])	2/304 (1% [0–2])	1/254 (0% [0–2])	1/224 (0% [0–3])	2/310 (1% [0–2])
Intermediate or posterior uveitis	0/310 (0% [0–1])	0/304 (0% [0–1])	0/254 (0% [0–2])	0/224 (0% [0–2])	0/310 (0% [0–1])
Involvement of the optic nerve†	0/308 (0% [0–1])	0/302 (0% [0–1])	0/252 (0% [0–2])	0/222 (0% [0–2])	0/308 (0% [0–1])
Functional outcome					
Optotype score <8/10	47/215 (22% [17–28])	41/214 (19 [14–25])	37/170 (22% [16–9])	32/143 (22% [16–30])	50/218 (23% [18–29])
Optotype score <3/10	3/215 (1% [0–4])	3/214 (1% [0–4])	3/170 (2% [0–6])	6/143 (4% [2–9])	7/218 (3% [1–7])

Data are n/N (% [95% CI]) unless otherwise specified. NA=not applicable.

* This includes findings at unplanned visits, not displayed in a separate column.

† Excluding two participants with a previous history of glaucoma and pallor of the optic nerve at enrolment, considered unrelated to mpox.
