# Supplementary material for: Prevalence, characteristics, and clinical course of mpox-related ophthalmic disease (MBOTE-EYE): a prospective cohort study in South Kivu, Democratic Republic of the Congo
Source: Lancet Glob Health. Author manuscript; Available in PMC 2026 Oct 9. (PMC13646179; doi:10.1016/j.langlo.2026.104054)
Supplement: Supplementary file 1 [file NIHMS2208071-supplement-1.pdf]

THE LANCET
Global Health

Supplementary appendix 1

This translation in French was submitted by the authors and we reproduce it as supplied. It has not been peer reviewed. *The Lancet*'s editorial processes have only been applied to the original in English, which should serve as reference for this manuscript.

Cette traduction en français a été proposée par les auteurs et nous l'avons reproduite telle quelle. Elle n'a pas été examinée par des pairs. Les processus éditoriaux du *Lancet* n'ont été appliqués qu'à l'original en anglais et c'est cette version qui doit servir de référence pour ce manuscrit.

Supplement to: Kabesha T, Van Dijck C, Mudwanga S, et al. Prevalence, characteristics, and clinical course of mpox-related ophthalmic disease (MBOTE-EYE): a prospective cohort study in South Kivu, Democratic Republic of the Congo. *Lancet Glob Health* 2026. https://doi.org/10.1016/j.langlo.2026.104054

Résumé

Contexte : Les données longitudinales issues d'un suivi ophtalmologique systématique des atteintes oculaires liées au mpox (MPOXROD) sont rares. Nous avons cherché à caractériser la prévalence et l'évolution clinique des MPOXROD dans le cadre d'une prise en charge symptomatique lors d'une flambée de mpox due au clade Ib.

Méthodes : MBOTE-EYE était une sous-étude intégrée à une cohorte prospective menée dans le Sud-Kivu, en République démocratique du Congo. Tous les patients hospitalisés présentant une infection à mpox confirmée par PCR au cours des 48 heures précédentes étaient éligibles. Les participants ont bénéficié d'un examen ophtalmologique à l'inclusion, à la sortie de l'hôpital, puis aux jours 29 et 59. Des écouvillons conjonctivaux ont été prélevés pour la détection du virus mpox par PCR. Les facteurs de risque de MPOXROD ont été évalués à l'aide de modèles de régression de Poisson à effets mixtes.

Résultats : Entre le 28 octobre 2024 et le 30 juin 2025, 310 participants ont été inclus (âge médian 14,0 ans [IQR 2,5–25,0] ; 166 [54 %] étaient de sexe féminin). À l'inclusion, une atteinte conjonctivale était présente chez 112 (36 % [IC à 95 % 31–42]) participants, une atteinte cornéenne chez 24 (8 % [5–11]) et une uvéite antérieure chez deux (1 % [0–2]). Au total, 134 (43 % [38–49]) participants ont développé une MPOXROD au cours du suivi, le plus souvent de manière bilatérale. Une cécité secondaire à une kératite ulcéreuse est survenue chez deux (1 % [0–3]) des 225 participants évaluables. Les lésions périorbitaires (rapport de risque ajusté [RRa] 3,65 [2,42–5,49]) et la malnutrition aiguë sévère (RRa 3,16 [1,22–8,22]) étaient associées à la survenue d'une MPOXROD. Une PCR conjonctivale a été réalisée chez 89 patients ; des valeurs de cycle seuil (Ct) inférieures à 25 n'ont été observées qu'en présence d'une MPOXROD active.

Interprétation : Les atteintes ophtalmologiques au cours du mpox de clade Ib sont fréquentes et souvent bilatérales, avec une fréquence importante de kératites et un risque de perte visuelle. Des examens ophtalmologiques systématiques devraient être intégrés à la prise en charge du mpox, et des traitements ophtalmologiques ciblés devraient être développés.
