# Supplementary material for: Prevalence, characteristics, and clinical course of mpox-related ophthalmic disease (MBOTE-EYE): a prospective cohort study in South Kivu, Democratic Republic of the Congo
Source: Lancet Glob Health. Author manuscript; Available in PMC 2026 Oct 9. (PMC13646179; doi:10.1016/j.langlo.2026.104054)
Supplement: Supplementary file 2 [file NIHMS2208071-supplement-2.pdf]

THE LANCET
Global Health

Supplementary appendix 2

This translation in Lingala was submitted by the authors and we reproduce it as supplied. It has not been peer reviewed. The Lancet's editorial processes have only been applied to the original in English, which should serve as reference for this manuscript.

Bolimboli oyo na lingala ekotisamaki na bakomi pe biso to bandeli bokotisi lolenge elekelaki. Bolimboli oyo etalamaki te na ba ninga misusu. Ba makambo oyo ya boponomi ba Lancet etalisamaki te na Mukanda ya yambo na anglais oyo esengelaki kozala lokola ndakisa pona Mukanda ya manuscrit oyo.

Supplement to: Kabesha T, Van Dijck C, Mudwanga S, et al. Prevalence, characteristics, and clinical course of mpox-related ophthalmic disease (MBOTE-EYE): a prospective cohort study in South Kivu, Democratic Republic of the Congo. *Lancet Glob Health* 2026. https://doi.org/10.1016/j.langlo.2026.104054

LINGALA

Na mokuse

Likambo ya koyekola Mikanda oyo elobeli bolandeli ya maladi ya miso oyo eutaka na mpox, oyo minganga ya miso balandaka na ndenge ya molai mpe ya ebongiseli (MPOXROD), ezali moke. Boyekoli oyo ezalaki na mokano ya koyeba bonene ya maladi yango mpe ndenge oyo ekobandaka mpe ekotambolaka na bato bazali kozwa lisalisi ya bokono na ntango ya bopanzani ya mpox ya clade Ib.

Ndenge boyekoli esalemaki MBOTE-EYE ezalaki boyekoli moko ya moke kati na boyekoli monene oyo ezalaki kolanda bato na ntango, na Sud-Kivu, na République Démocratique du Congo. Bato nyonso oyo balalisamaki na lopitalo mpe bazwamaki na mpox oyo endimami na ekzamɛ ya PCR (na kati ya ngonga 48 eleki) bakokaki kokɔta na boyekoli. Minganga ya miso batalaki miso ya bato wana na ebandeli, tango bazalaki kobima na lopitalo, mpe lisusu na mokolo ya 29 mpe ya 59. Bazwaki mpe échantillon na conjonctive mpo na koluka virusi ya mpox na PCR. Makambo oyo ekoki kobakisa likama ya kozwa MPOXROD etalamaki na nzela ya analyse statistique ya Poisson na effets mixtes.

Makambo oyo emonanaki Kobanda mokolo ya 28 sanza ya zomi ya mobu 2024 kino mokolo ya 30 sanza ya motoba ya mobu 2025, bato 310 bakɔtaki na boyekoli (mbula ya katikati 14 [IQR 2,5–25,0] ; bato 166 [54 %] bazalaki basi). Na ebandeli ya boyekoli, bato 112 (36 % [IC 95 % 31–42]) bazalaki na maladi ya conjonctive, bato 24 (8 % [5–11]) bazalaki na maladi ya cornée, mpe bato mibale (1 % [0–2]) bazalaki na uvéite antérieure. Na mobimba, bato 134 (43 % [38–49]) bazwaki MPOXROD na ntango ya bolandeli, mpe mbala mingi maladi yango ezalaki na miso nyonso mibale. Bato mibale kati na 225 (1 % [0–3]) bakufelaki komona mpo na kératite ulcéreuse. Bato oyo bazalaki na baplai pembeni ya miso (RRa 3,65 [2,42–5,49]) mpe baoyo bazalaki na malnutrition aiguë sévère (RRa 3,16 [1,22–8,22]) bazalaki na likama monene ya kozwa MPOXROD. Ekzamɛ ya PCR na conjonctive esalemaki na bato 89, mpe valeur ya Ct oyo ezalaki na se ya 25 emonanaki kaka epai ya bato oyo bazalaki na MPOXROD oyo ezalaki naino na phase ya makasi.

Ndimbola Maladi ya miso oyo etali mpox ya clade Ib ezali mingi mpe mbala mingi ekangaka miso nyonso mibale. Ezali kobimisa mingi maladi ya cornée mpe ekoki komema na kobungisa komona. Yango wana, kotala miso na ndenge ya ebongiseli esengeli kokɔtisama na lisalisi ya bato bazali na mpox, mpe esengeli kosala bolukiluki mpo na kozwa bankisi ya maladi ya miso oyo ebongisami mpo na bokono yango.
