# Supplementary material for: Prevalence, characteristics, and clinical course of mpox-related ophthalmic disease (MBOTE-EYE): a prospective cohort study in South Kivu, Democratic Republic of the Congo
Source: Lancet Glob Health. Author manuscript; Available in PMC 2026 Oct 9. (PMC13646179; doi:10.1016/j.langlo.2026.104054)
Supplement: Supplementary file 3 [file NIHMS2208071-supplement-3.pdf]

THE LANCET
Global Health

Supplementary appendix 3

This translation in Swahili was submitted by the authors and we reproduce it as supplied. It has not been peer reviewed. *The Lancet*'s editorial processes have only been applied to the original in English, which should serve as reference for this manuscript.

Tafsiri hii katika Swahili iliwasilishwa na waandishi na tunatengeneza tena kama hutolewa. Haijapitiwa. Mchakato wa hariri wa Lancet umetumika tu kwa asili kwa Kiingereza, ambayo inapaswa kutumika kama kumbukumbu kwa muswada hii.

Supplement to: Kabesha T, Van Dijck C, Mudwanga S, et al. Prevalence, characteristics, and clinical course of mpox-related ophthalmic disease (MBOTE-EYE): a prospective cohort study in South Kivu, Democratic Republic of the Congo. *Lancet Glob Health* 2026. https://doi.org/10.1016/j.langlo.2026.104054

Kiwango cha maambukizi, sifa, na maendeleo ya alama za ugonjwa wa macho unaohusiana na mpox (MBOTE-EYE): utafiti wa kundi tarajiwa katika Kivu kusini, Jamhuri ya Kidemokrasia ya Kongo.

Muhtasari:

Usuli: utafiti tathmini ya kitaratibu ya mtaalamu wa macho inayoongozwa na daktari wa macho ya ugonjwa wa macho unaohusiana na mpox(MPOXROD) imeripotiwa kwa hakika. Tulilenga kubainisha maambukizi na maendeleo ya alama za ugonjwa wa MPOXROD chini ya usimamizi shirikishi wakati wa mlipuko wa clade Ib.

Method: MBOTE-EYE ilikuwa utafiti mdogo uliowekwa ndani ya kundi linalotarajiwa huko Kivu Kusini, Jamhuri ya Kidemokrasia ya Kongo. Wagonjwa wote waliolazwa hospitalini walio na mpox iliyothibitishwa na PCR (masaa 48 zilizopita) walistahiki. Wagonjwa walioshiriki utafiti huu walipimwa macho tangu kuorozeshwa kwao, na kutoshwa hospital, na siku 29 na 59 baada ya kutoka hospital. Vipu vya kope la ndani vilikusanywa kwa PCR ya virusi vya mpox. Sababu za hatari kwa MPOXROD zilitathminiwa kwa kutumia marejeleo ya Poisson ya athari mchanganyiko.

Matokeo: kati ya Oct28,24 na Juni30,2025, washiriki 310 wali orodheshwa na fwatliliwa. Umri wa wastani ulikuwa miaka 14,0 (IQR 2.5-25.0; [54%] walikuwa wanawake). Wakati wa kuorozeshwa, wagonjwa 112 (36%[95% CI 312] walikuwa na ugonjwa wa kope la ndani la jicho (macho mekundu). Wengine 24(8%[(5-11)]) walikuwa na ugonjwa wa kornea(vyoo vya macho), na wawili, walikuwa na uveitis ya mbele (1%[0-2]. Kwa ujumla washiriki 134 (43%[38-49] walipata MPOXROD wakati wa ufuatiliaji, na macho yote mawili yali husika kwa wengi kati yao. wagonjwa wawili (1%[0-3] walipofuka kutokana na vidonda vya kornea (juu ya vyoo vya macho). kati ya wagonjwa 225.

Vidonda vya eneo linalozunguka jicho. uwiano[aRR] 3.65 [2.42-5.49]) na utapiamlo mkali wa papo hapo (aRR 3.16[1.22-8.22]) vilihusishwa na MPOXROD. Vipimo PCR vilifanyika kwa

wagonjwa 89 na viwango vya chini vya mzunguko chini ya 25 vilitokea pekee wakati wa MPOXROD amilifu.

Ufafanuzi Kuhusika kwa macho katika clade Ib mpox ni jambo la kawaida na mara nyingi lina husu macho yote mawili, lenye mzigo mkubwa wa keratiti na hatari ya kupoteza uwezo wa kuona. Uchunguzi wa kimfumo unapaswa kuongezwa katika matunzo ya mpox, na matibabu yaliyolengwa ya macho yanapaswa kuendelezwa.
