# Supplementary material for: Prevalence, characteristics, and clinical course of mpox-related ophthalmic disease (MBOTE-EYE): a prospective cohort study in South Kivu, Democratic Republic of the Congo
Source: Lancet Glob Health. Author manuscript; Available in PMC 2026 Oct 9. (PMC13646179; doi:10.1016/j.langlo.2026.104054)
Supplement: Supplementary file 4 [file NIHMS2208071-supplement-4.pdf]

THE LANCET
Global Health

Supplementary appendix 4

This appendix formed part of the original submission and has been peer reviewed.
We post it as supplied by the authors.

Supplement to: Kabesha T, Van Dijck C, Mudwanga S, et al. Prevalence, characteristics, and clinical course of mpox-related ophthalmic disease (MBOTE-EYE): a prospective cohort study in South Kivu, Democratic Republic of the Congo. *Lancet Glob Health* 2026. https://doi.org/10.1016/j.langlo.2026.104054

Appendix to Manuscript "Prevalence, Characteristics and Clinical Course of Mpox-Related Ophthalmic Disease: A Prospective Cohort Study in South-Kivu, Democratic Republic of the Congo (MBOTE-EYE)"

Table of Contents

Figure S1: Risk factors for mpox-related ophthalmic disease: directed acyclic graph 3

Figure S2: Mpox-related ophthalmic disease features in different individuals 4

Table S1: Presence of ophthalmic signs and symptoms, per participant 5

Table S2: Prevalence of eye disease, per participant, by age category. 7

Table S3: Topical treatments prescribed throughout the study 8

Table S4: Result of MPXV-PCR on conjunctival swabs, by eye, at presentation and during follow-up 9

Table S5: Risk factors for mpox-related ophthalmic disease 10

Study protocol 11

Figure S1: Risk factors for mpox-related ophthalmic disease: directed acyclic graph

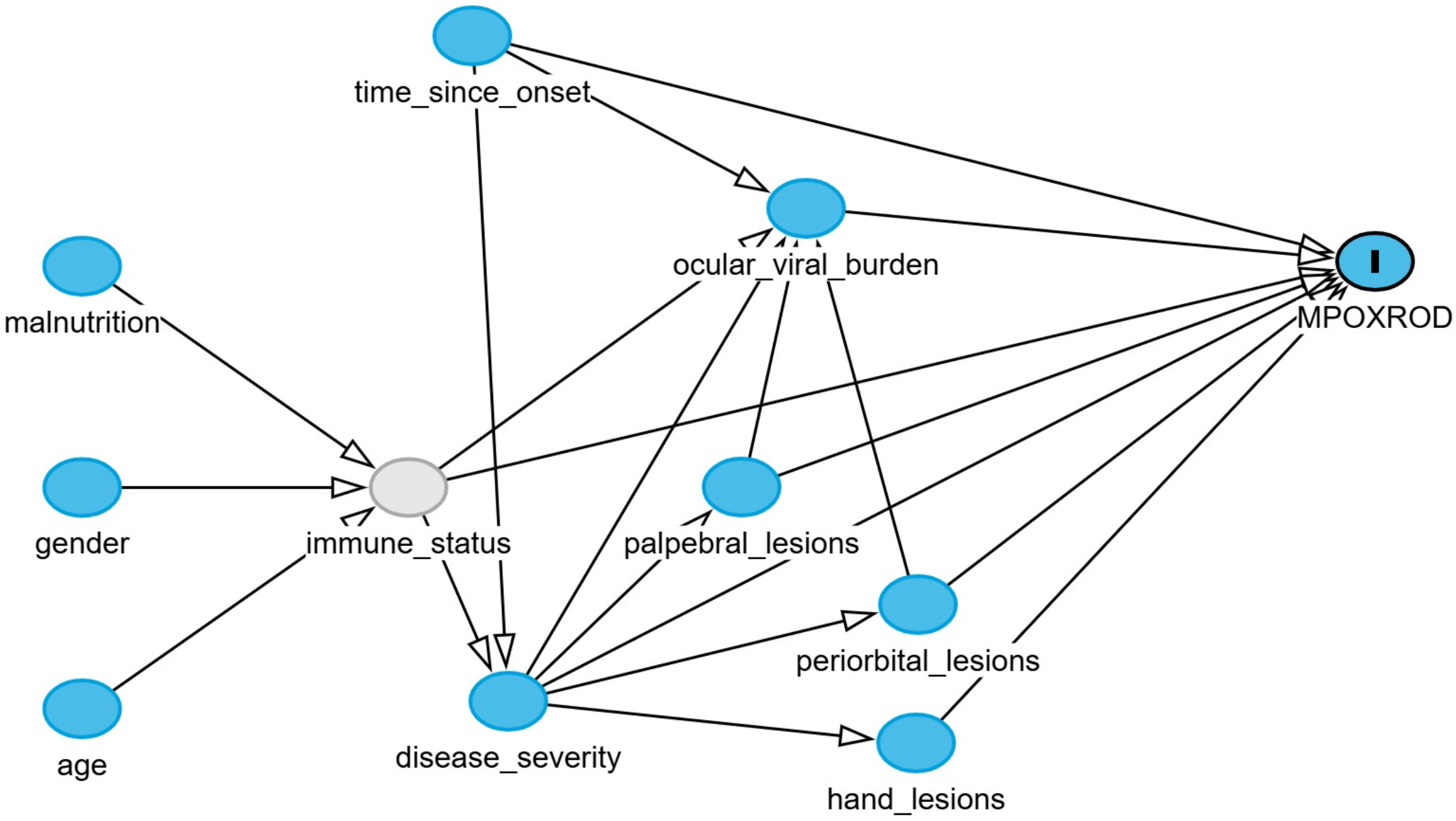

Generated using DAGitty: Johannes Textor, Benito van der Zander, Mark K. Gilthorpe, Maciej Liskiewicz, George T.H. Ellison. Robust causal inference using directed acyclic graphs: the R package 'dagitty'. International Journal of Epidemiology 45(6):1887-1894, 2016.

Figure S2: Mpox-related ophthalmic disease features in different individuals

(A) Left eye: extensive periorbital and palpebral mpox lesions, healing over time;
(B) Right eye: extensive periorbital and palpebral mpox lesions with blepharitis;
(C) Left eye: mpox lesion on the palpebral mucosa of the upper eyelid over time;
(D) Right eye: ulcerative keratitis;
(E) Right eye: blepharitis with palpebral mpox lesion, purulent secretions, ulcerative keratitis over time;
(F) Uveitis with hypopyon and endophthalmitis (visual acuity: light perception only), with improvement over time (visual acuity: 2/10);
(G) blepharitis, conjunctivitis, superficial keratitis evolving into corneal opacification (scarring).

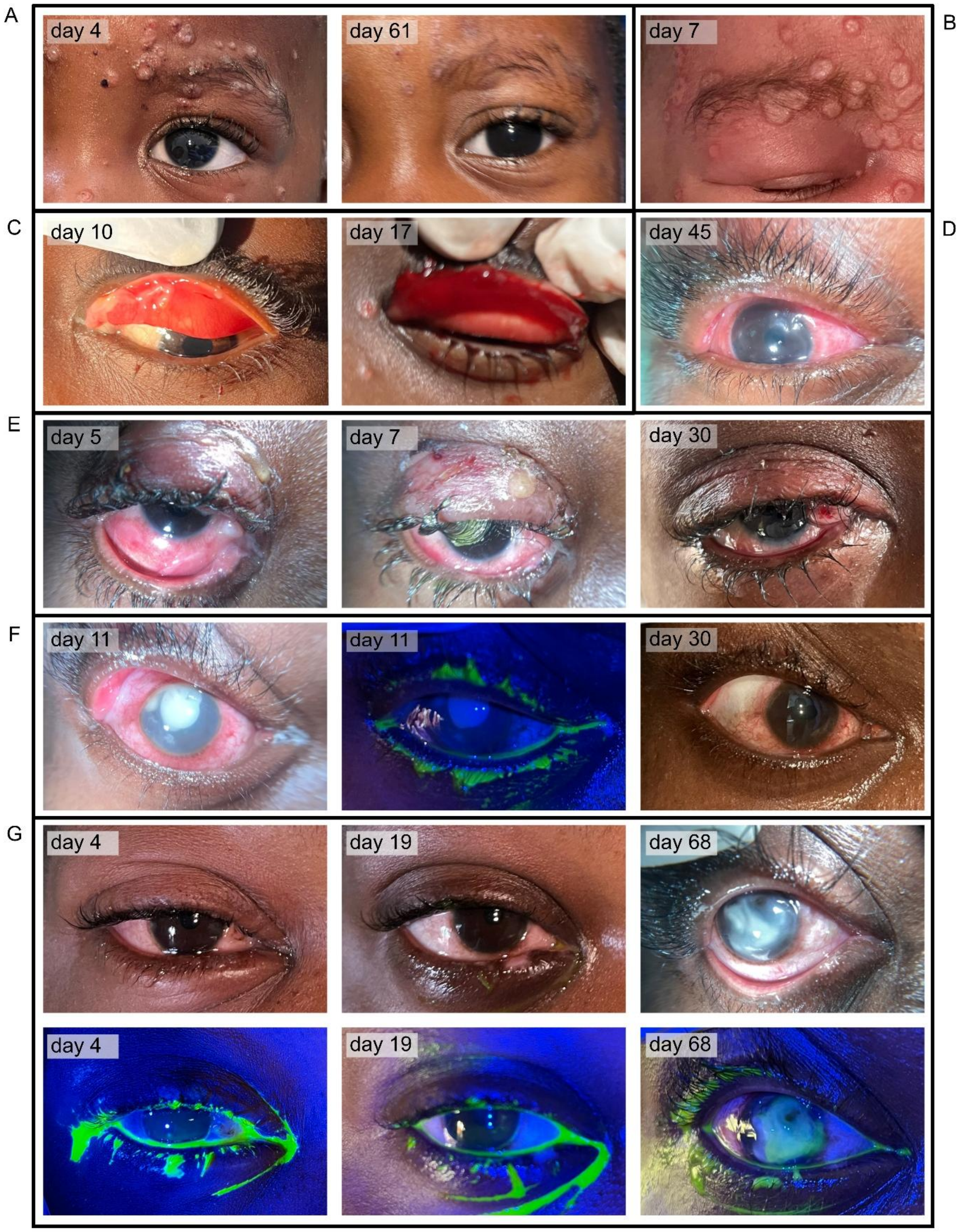

Table S1: Presence of ophthalmic signs and symptoms, per participant

	Inclusion (N=310)	Discharge (N=304)	Day 29 (N=254)	Day 59 (N=224)	Any time point* (N=310)
**Number of days post onset of symptoms, median, (IQR)**	6·0 (4·0–8·0)	16·0 (13·0–22·0)	34·0 (32·0–36·0)	64·0 (62·0–66·0)	NA
**Ophthalmic symptoms (n/N, %)**	154/310 (50)	34/304 (11)	39/254 (15)	22/224 (10)	172/310 (56)
Itchiness	64/310 (21)	6/304 (2)	12/254 (5)	10/224 (45)	79/310 (26)
Photophobia	62/310 (20)	9/304 (3)	11/254 (4)	4/224 (2)	75/310 (24)
Redness	39/310 (13)	7/304 (2)	8/254 (3)	5/224 (2)	46/310 (15)
Pain	36/310 (12)	3/304 (1)	5/254 (2)	3/224 (1)	42/310 (14)
Discharge or pseudo membranes	31/310 (10)	1/304 (0)	0/254 (0)	3/224 (1)	34/310 (11)
Decreased visual acuity	24/310 (8)	15/304 (5)	9/254 (4)	7/224 (3)	29/310 (9)
Tearing	17/310 (6)	4/304 (1)	4/254 (2)	4/224 (2)	26/310 (8)
Feeling of foreign object	10/310 (3)	2/304 (1)	3/254 (1)	3/224 (1)	16/310 (5)
Dryness	3/310 (1)	1/304 (0)	0/254 (0)	0/224 (0)	5/310 (2)
Vision loss	0/310 (0)	0/304 (0)	0/254 (0)	2/224 (1)	2/310 (1)
Other	13/310 (4)	1/304 (0)	2/254 (1)	1/224 (0)	18/310 (6)
**Abnormal eye motility, n/N (%)**	0/310 (0)	1/304 (0)	1/254 (0)	0/224 (0)	2/310 (1)
**Mpox lesions**					
Periorbital, n/N (%)	78/310 (25)	0/304 (0)	0/254 (0)	0/224 (0)	79/310 (26)
On the palpebra, n/N (%)	60/310 (19)	1/304 (0)	0/254 (0)	1/224 (0)	66/310 (21)
On the conjunctiva, n/N (%)	10/310 (3)	4/304 (1)	3/254 (1)	1/224 (0)	13/310 (4)
**Palpebral edema, n/n (%)**	18/310 (6)	4/304 (1)	3/254 (1)	3/224 (1)	23/310 (7)
**Periorbital edema, n/n (%)**	0/310 (0)	0/304 (0)	0/254 (0)	0/224 (0)	0/310 (0)
**Conjunctival abnormality, n/N (%)**	112/310 (36)	20/304 (7)	26/254 (10)	14/224 (6)	132/310 (43)
Conjunctivitis, n/N (%)	108/112 (96)	16/20 (80)	23/26 (89)	13/14 (93)	127/132 (96)
Mild, n/N (%)	81/106 (76)	15/15 (100)	18/22 (78)	11/13 (82)	106/127 (84)
Severe, n/N (%)	25/106 (24)	0/15 (0)	4/22 (18)	2/13 (15)	29/127 (23)
Conjunctival mpox lesions, n/N (%)	10/112 (9)	4/20 (20)	3/26 (12)	1/14 (7)	13/132 (10)
**Scleritis, n/N (%)**	0/119 (0)	0/304 (0)	0/254 (0)	0/224 (0)	0/310 (0)
**Corneal abnormality, n/N (%)**	24/310 (8)	9/304 (3)	8/254 (3)	4/224 (2)	29/310 (9)
Corneal opacity or scarification, n/N (%)	2/24 (8)	0/9 (0)	0/8 (0)	2/4 (50)	4/29 (14)
Corneal epithelial defect or ulcer, n/N (%)	22/24 (92)	9/9 (100)	8/8 (100)	2/4 (50)	27/29 (93)
Central, n/N (%)	12/22 (55)	3/9 (38)	1/8 (13)	2/2 (100)	13/27 (48)
Peripheral, n/N (%)	16/22 (73)	6/9 (75)	7/8 (88)	0/1 (0)	24/27 (89)
Corneal edema, n/N (%)	0/24 (0)	0/9 (0)	0/8 (0)	1/4 (25)	1/29 (3)
**Hypopion, n/N (%)**	1/310 (0)	0/304 (0)	0/254 (0)	1/224 (0)	2/310 (1)
**Abnormality of the iris, n/N (%)**	2/310 (1)	2/304 (1)	1/254 (0)	1/224 (0)	2/310 (1)
Synechiae, n/N (%)	1/2 (50)	1/2 (50)	1/1 (100)	1/1 (100)	1/2 (50)

	Inclusion (N=310)	Discharge (N=304)	Day 29 (N=254)	Day 59 (N=224)	Any time point* (N=310)
Transillumination defects, n/N (%)	1/2 (50)	1/2 (50)	0/1 (0)	0/1 (0)	1/2 (50)
**Abnormality of the pupil, n/N (%)**	1/310 (0)	1/304 (0)	0/254 (0)	2/224 (1)	3/310 (1)
Semi-mydriasis or Mydriasis, n/N (%)	0/1 (0)	0/1 (0)	0/0 (NA)	1/2 (50)	1/3 (33)
Sluggish or absent photomotor reflex, n/N (%)	1/1 (100)	1/1 (100)	0/0 (NA)	1/2 (50)	2/3 (67)
**Raised intraocular pressure (Tonopen value > 21 mmHg), n/N (%)**	1/310 (0)	0/304 (0)	0/254 (0)	0/224 (0)	1/310 (0)
**Cataract, n/N (%)**	5/310 (2)	3/304 (1)	3/254 (1)	2/224 (1)	5/310 (2)
**Any vitreous abnormality, n/N (%)**	1/310 (0)	0/304 (0)	0/254 (0)	1/224 (0)	2/310 (1)
Opaque vitreous humor, n/N (%)	1/1 (100)	0/0 (NA)	0/0 (NA)	1/1 (100)	2/2 (100)
**Any abnormality of the optic nerve, n/N (%)**	2/310 (1)	2/304 (1)	2/254 (1)	0/224 (0)	2/310 (1)
Optic nerve pallor, n/N (%)	2/2 (100)	2/2 (100)	2/2 (100)	0/0 (NA)	2/2 (100)
**Any macular abnormality, n/N (%)**	0/310 (0)	0/304 (0)	0/254 (0)	0/224 (0)	0/310 (0)
**Any retinal vascular abnormality, n/N (%)**	0/310 (0)	0/304 (0)	0/254 (0)	0/224 (0)	0/310 (0)
**Any peripheral retinal abnormality, n/N (%)**	0/310 (0)	0/304 (0)	0/254 (0)	0/224 (0)	0/310 (0)
**Visual acuity (far sight, without correction), n/N (%)**					
Optotype chart used	215/310 (69)	214/304 (70)	170/254 (67)	143/224 (64)	218/310 (70)
< 1/20	2/215 (1)	1/214 (1)	1/170 (1)	3/143 (2)	4/218 (2)
[1/20 - 1/10[	0/215 (0)	0/214 (0)	0/170 (0)	0/143 (0)	2/218 (1)
[1/10 - 3/10[	1/215 (1)	2/214 (1)	2/170 (1)	3/143 (2)	6/218 (3)
[3/10 - 8/10[	44/215 (21)	38/214 (18)	34/170 (20)	26/143 (18)	47/218 (22)
[8/10 - 10/10]	168/215 (78)	173/214 (81)	133/170 (78)	111/143 (78)	177/218 (81)
Impossibility to use optotype chart	95/310 (31)	90/304 (30)	84/254 (33)	81/224 (36)	308/310 (99)
Good light tracking	95/95 (100)	90/90 (100)	83/83 (100)	81/81 (100)	99/99 (100)
**Visual acuity (far sight, with correction), n/N (%)**					
Optotype chart used	8/310 (3)	4/304 (1)	2/254 (1)	0/224 (0)	10/310 (3)
[3/10 - 8/10[	0/8 (0)	1/4 (25)	0/2 (0)	0/0 (NA)	1/10 (10)
[8/10 - 10/10]	8/8 (100)	3/4 (75)	2/2 (100)	0/0 (NA)	9/10 (90)

*This includes findings at unplanned visits, not displayed in a separate column

Table S2: Prevalence of eye disease, per participant, by age category.

	Age < 5 years	Age 5 – 14 years	Age > 14 years
**Affection of periorbita & palpebra, n/N (%; 95% CI)**			
Periorbital mpox lesions	35/105 (33)	14/56 (25)	30/149 (20)
Blepharitis	12/105 (11)	4/56 (7)	8/149 (5)
Palpebral mpox lesions	31/105 (30)	10/56 (18)	25/149 (17)
**MPOXROD (mpox-related ophthalmic disease), n/N (%)**	65/105 (62)	20/56 (36)	49/149 (33)
Conjunctival disease	62/105 (59)	20/56 (36)	50/149 (34)
Conjunctivitis without conjunctival mpox lesions	59/62 (95)	17/20 (85)	43/50 (86)
Conjunctival mpox lesion(s) with or without conjunctivitis	3/62 (5)	3/20 (15)	7/50 (14)
Scleritis	0/105 (0)	0/56 (0)	0/149 (0)
Corneal disease	16/105 (15)	3/56 (5)	10/149 (7)
Without epithelial defect	2/16 (13)	0/3 (0)	0/10 (0)
With epithelial defect	14/16 (88)	3/3 (100)	10/10 (100)
Anterior uveitis	0/105 (0)	0/56 (0)	2/149 (1)
Intermediate/posterior uveitis	0/105 (0)	0/56 (0)	0/149 (0)
Affection of the optic nerve	0/105 (0)	0/56 (0)	0/147 (0)$
**Functional outcome at day 59, n/N (%)**			
Optotype score < 8/10	13/105 (12)	14/56 (25)	23/149 (15)
Optotype score < 3/10	0/105 (0)	2/56 (4)	5/149 (3)

$ Excluding two participants with a prior history of glaucoma and pallor of the optic nerve at enrolment

Table S3: Topical treatments prescribed throughout the study

Medicine, n/N (%)	Overall (N = 310)	Keratitis (N = 29)	Conjunctivitis (N = 132)	Anterior uveitis (N = 2)
Antibiotic eye drops	88/310 (28)	27/29 (93)	75/132 (57)	0/2 (0)
Sodium cromoglycate eye drops	57/310 (18)	5/29 (17)	55/132 (42)	0/2 (0)
NSAID eye drops	52/310 (17)	23/29 (79)	42/132 (32)	0/2 (0)
Tetracycline ointment	43/310 (14)	21/29 (72)	33/132 (25)	0/2 (0)
Artificial tears	28/310 (9)	4/29 (14)	16/132 (12)	0/2 (0)
Prednisolone eye drops	24/310 (8)	4/29 (14)	22/132 (17)	2/2 (100)
Acyclovir eye drops	2/310 (1)	2/29 (7)	2/132 (2)	0/2 (0)
Atropine eye drops	2/310 (1)	1/29 (3)	1/132 (1)	2/2 (100)
Dexamethasone-gentamycin injection (sub-conjunctival)	2/310 (1)	1/29 (3)	1/132 (1)	1/2 (50)
Beta antagonist eye drops	2/310 (1)	0/29 (0)	2/132 (2)	0/2 (0)

Table S4: Result of MPXV-PCR on conjunctival swabs, by eye, at presentation and during follow-up

	Inclusion	Discharge	Day 29
**Number of participants eligible for conjunctival swab collection***	89	59	16
**Number of participants sampled (% of those eligible)**	89 (100)	53 (90)	15 (94)
**Number of available swabs**	178	106	30
**Number of valid test results (% of available swabs)**	178 (100)	104 (98)	30 (100)
**Proportion testing positive, n/N (%)**	132/178 (74)	65/104 (63)	9/30 (30)
**Median ROX Ct value (IQR)**	33.4 (29.7 – 40.0)	35.7 (33.2 – 40.0)	40.0 (37.0 – 40.0)
**Median ROX Ct value (IQR) among PCR-positive samples**	31.7 (28.9 – 34.7)	33.8 (32.7 – 35.3)	35.0 (34.7 – 36.4)

*Conjunctival swabs were collected from consecutive study participants attending study visits between 29 October 2024 and 5 December 2024

Table S5: Risk factors for mpox-related ophthalmic disease

Risk factor	Multivariable regression model adjusted for	aRR (95% CI)
**Age group**	Gender, malnutrition, time since symptom onset	
> 14 years		*Reference category*
5-14 years		0.98 (0.58–1.64)
< 5 years		**1.95 (1.28–2.98)**
**Gender**	Age group, malnutrition, time since symptom onset	
Women		*Reference category*
Man		1.09 (0.75–1.59)
**Acute malnutrition£**	Age group, gender, time since symptom onset	
No/moderate		*Reference category*
Severe		**3.16 (1.22–8.22)**
**Mpox lesion distribution**		
Presence of palpebral mpox lesions	Age group, gender, malnutrition, time since symptom onset	**2.10 (1.50–2.94)**
Presence of periorbital mpox lesions	Age group, gender, malnutrition, time since symptom onset	**3.65 (2.42–5.49)**
Presence of mpox lesions on the hands	Age group, gender, malnutrition, time since symptom onset	0.81 (0.54–1.21)
**Lesion severity**	Age group, gender, malnutrition, time since symptom onset	
Mild (<25)		*Reference category*
Moderate (25-99)		0.84 (0.49–1.44)
Severe (100-250)		1.05 (0.64–1.73)
Grave (>250)		1.10 (0.66–1.9)
**Viral burden**	Age group, gender, time since symptom onset	
Swab PCR positivity		**0.44 (0.22–0.85)**
Inverted Ct-value of PCR-positive swabs *		**1.12 (1.04–1.19)**

£ Moderate acute malnutrition was defined as MUAC 11.5–12.5 cm in children <5 years; BMI-for-age z-score −3 to −2 SD in participants aged 5–19 years; and BMI 16–18 kg/m² in adults >19 years. Severe acute malnutrition was defined as MUAC <11.5 cm in children <5 years; BMI-for-age z-score <−3 SD in participants aged 5–19 years; and BMI <16 kg/m² in adults >19 years.

* represented by the interaction term between inverted Ct-values and PCR positivity

Study protocol

Research Study Protocol

MAPPING THE BURDEN OF OCULAR COMPLICATIONS OF MPOX IN DRC

(MPOX-EYE STUDY)

Version 2.0, July 15, 2024

Partners:

**SPONSOR**	Institut National de la Recherche Biomédicale (INRB) Democratic Republic of the Congo (DRC)<br>Institute of Tropical Medicine (ITM), Belgium
**FUNDER**	Fondation Flandre pour la recherche (FWO)
**PARTNERS**	Institut National de la Recherche Biomédicale (INRB) Democratic Republic of the Congo (DRC)<br>National Institute of Allergy and Infectious Diseases (NIAID)<br>Institute of Tropical Medicine (ITM), Belgium

**COORDINATING INVESTIGATOR INRB:**	Prof. dr. Placide Mbala- Kingebeni
**DEPARTMENT:**	Epidemiology and Global Health
**ADDRESS:**	Av. De la Démocratie N°5345, Kinshasa – Gombe, the Democratic Republic of the Congo
**TELEPHONE:**	+243822851584
**EMAIL:**	mbalaplacide@gmail.com

**COORDINATING INVESTIGATOR ITM:**	Prof. dr. Laurens Liesenborghs
**DEPARTMENT:**	Clinical sciences
**ADDRESS:**	Nationalestraat 155, 2000 Antwerpen, Belgium
**TELEPHONE:**	+32475311904
**EMAIL:**	lliesenborghs@itg.be

**Protocol History**		
**Protocol version/ Amendment**	**Date**	**Reason for amendment**
Original version (1.0)	06 February 2024	NA

Revised version (1.1)	19 March 2024	Revision based on ITM IRB comments
Amended version (2.0)	15 July 2024	Addition of study site “Kamituga general hospital”

1 Protocol Synopsis

<table>
<tr><td>Rationale</td><td>Clade I monkeypox virus (MPXV) infection in Central Africa is often associated with complications including bacterial skin infection, eye infection, bacteraemia, sepsis, encephalitis, and death. Eye complications appear to be particularly frequent as about 20% of mpox patients are estimated to suffer ocular complications that can lead to blindness. Conjunctivitis and corneal lesions are described among patients with clade IIb MPXV infection in Europe and North America. However, descriptive studies of ocular complications during and after mpox in endemic regions are insufficiently documented and outdated. There is a pressing need to map the actual burden of acute and chronic ocular complications of mpox infections in endemic regions to guide service provision to the affected individuals and populations.</td></tr>
<tr><td>Study aim</td><td>Describe the frequency and clinical characteristics of the ocular manifestations of mpox during acute infection and convalescence.</td></tr>
<tr><td>Design</td><td>Prospective observational cohort study of patients infected with MPXV.</td></tr>
<tr><td>Study site & population</td><td>The study will take place in parallel to:
- the PALM007 clinical trial, which evaluates the efficacy of tecovirimat for the treatment of mpox in two sites in the Democratic Republic of the Congo: Tunda General Hospital, Maniema Province and Kole General Hospital, Sankuru Province.
- The MBOTE study, which evaluates the clinical signs and symptoms in people with mpox at Kamituga General Hospital, South Kivu Province.

Depending on the mpox epidemiological context other sites can be included at a later timepoint.

Participants will be recruited among PCR-confirmed mpox cases, including but not limited to patients enrolled in the PALM 007 trial and MBOTE study. The study may be continued after cessation of the PALM007 trial and MBOTE study.</td></tr>
<tr><td>Inclusion & Exclusion criteria</td><td><u>Inclusion criteria</u>
▪ Patient of any age or sex with laboratory-confirmed infection with MPXV
▪ Laboratory confirmation of MPXV infection less than 48 hours before inclusion
▪ Patient or culturally acceptable representative is willing and able to give informed consent or assent for participation in the study

<u>Exclusion criteria</u>
▪ Patients for whom, in the judgment of the recruiting physician, participation in the study would interfere with routine optimal clinical care.</td></tr>
</table>

**OBJECTIVES AND OUTCOMES**	**Objectives**	**Outcome measure**
	**Primary objective**	
	To determine the prevalence of **eye disease and visual deficiencies** at presentation	Composite presence or absence of:<br>• orbital lesions (exophthalmos, enophthalmos, oculomotor paralysis...)<br>• affection of the adnexa<br>• affection of the anterior segment<br>• affection of the posterior segment<br>• affection of the optic nerve
	**Secondary objectives**	
	To describe **all ocular symptoms and signs** at presentation and during follow-up	Presence or absence of individual symptoms and signs over time
	To assess **visual acuity** at presentation and during follow-up	Result of Optotype letters/numbers and/or E test over time
	To determine the **presence of MPXV in tear fluid** at presentation and during follow-up	Result of MPXV-PCR on conjunctival swabs over time
**SAMPLE SIZE**	As high as possible, depending on the evolution of the epidemic. A sample size of 62 patients would allow detection of a prevalence of 20% of acute complications with a confidence level of 95% and a margin of error of 10%. Recruiting 246 participants allows detection of the same prevalence with a margin of error of 5%.	
**STUDY PROCEDURES**	Study visits will be done:<br>▪ At diagnosis of mpox<br>▪ At follow-up day 14 post inclusion (+/- 3 days), only for hospitalized patients<br>▪ At follow-up day 29 post inclusion (+/- 7 days)<br>▪ At follow-up day 59 post inclusion (+/- 7 days)<br>▪ During unscheduled visits between enrolment and the end-of-study visit (day 59) upon presentation of new or worsening ocular symptoms based on a 3-point checklist.<br><br>During study visits, the following procedures will be performed:<br>▪ Questionnaire concerning demographics, relevant medical history, past and current eye symptoms	

	▪ Clinical ophthalmological assessment including:<br>○ Evaluation of visual acuity (optotype test)<br>○ Evaluation of pupillary light reflex and eye movements<br>○ Examination and photographic imaging of the anterior segment using slit lamp examination before and after application of fluoresceine<br>○ Evaluation of quantitative (Schirmer test) and qualitative (Break up time) tear secretions<br>○ Dilated fundoscopic exam and photographic imaging of the retina<br>▪ Conjunctival swab<br><br>If patients are hospitalized, the following checklist will be asked daily by an attending health care worker:<br><br>▪ new or worsening reduced visual acuity ? Yes/no<br>▪ new or worsening photophobia? Yes/no<br>▪ new or worsening significant eye pain? Yes/no<br>▪ new or worsening exterior eye-lid lesion or edema? Yes/no<br><br>If the answer to any of the checklist questions is “Yes”, an unscheduled visit will be performed for a more thorough ophthalmological assessment.<br><br>If patients are not hospitalized or after discharge from the hospital, patients are encouraged to attend for an unscheduled visit if any of the checklist-symptoms occur or worsen.
**LABO ANALYSES**	▪ PCR for MPXV on conjunctival swabs with tear fluid
**TIMELINES**	▪ Submission EC: 31 July 2024<br>▪ Study start: September 2024<br>▪ Duration: 1 year
**PARTNERS**	▪ Institut National de la Recherche Biomédicale (INRB)<br>▪ Institute of Tropical Medicine (ITM)<br>▪ National Institute of Allergy and Infectious Diseases (NIAID)

2 TABLE OF CONTENTS

1 Protocol Synopsis 4

2 Table of Contents 7

3 Statement of Compliance 9

4 Introduction 10

4.1 Background and rationale 10

5 Study Objectives and Outcomes 12

6 Study Design 12

7 Methods 12

7.1 Study Setting, Population and Sampling Strategy 12

7.1.1 Sample Size and Power 12

7.1.2 Inclusion Criteria 13

7.1.3 Community participation 13

7.2 Procedures 13

7.2.1 General overview of study visits 13

7.2.2 Procedures performed 13

7.2.3 End of study 14

7.2.4 Laboratory Procedures 15

7.2.5 Biobanking 16

7.3 Data Analysis 17

7.3.1 Quantitative Data Analysis 17

8 Ethical Issues 17

8.1 Ethical (and regulatory) Review 17

8.2 Obtaining Informed Consent 17

8.3 Assent Process 18

8.4 Risks and Benefits of Participation 18

8.5 Vulnerable populations 19

8.5.1 Impoverished people 19

8.5.2 Children 19

8.5.3 Pregnant and lactating women 19

8.6 Insurance 19

8.7 Confidentiality and privacy 19

9 Monitoring And Quality Control 20

10 Timelines *20*

11 Collaborative partnership *20*

12 Data Management And Archiving *21*

12.1 Data Management Responsibilities 21

12.2 Data Collection Methods 21

12.3 Data Security And Confidentiality 21

12.4 Data Sharing 22

12.5 Archiving 22

13 Dissemination of Results *22*

14 List Of Abbreviations *22*

15 Annexes *23*

16 References *23*

3 STATEMENT OF COMPLIANCE

This protocol contains the necessary information for conducting this research study. By signing this document, the Investigator commits to carry out the study in compliance with the protocol, the applicable ethical guidelines like the Declaration of Helsinki, the European General Data Protection Regulation (GDPR), the ESF/ALLEA Code of Conduct for Research Integrity, and consistent with international scientific standards as well as all applicable regulatory requirements. The Investigator will also make every reasonable effort to complete the study within the timelines designated.

Once the final protocol has been issued and signed by the Investigator(s) and the authorized signatories, it cannot be informally altered. Protocol amendments have the same legal status and must pass through the mandatory steps of review and approval before being implemented.

Principal Investigator INRB:

Title, Name: prof. Placide Mbala-Kingebeni Date: 15 July 2024

Signed:

Principal Investigator ITM:

Title, Name: prof. Laurens Liesenborghs Date: 15 July 2024

Signed:

4 INTRODUCTION

4.1 BACKGROUND AND RATIONALE

General introduction

Mpox (formerly monkeypox), caused by monkeypox virus (MPXV) infection, caught the attention of the global health community during an unprecedented global outbreak in 2022. Long before this outbreak however, the geographic burden and incidence of mpox was notably increasing in several African countries, particularly in the Democratic Republic of the Congo (DRC), which has noted more than a ten-fold increase in annual cases, from the 1990s (≈500 cases/yr) to 2022 (5.671 cases) including in previously unaffected provinces (source: DRC Ministry of Health). As opposed to the 2022 global outbreak (related to clade II MPXV), mpox in the DRC (so far only caused by clade I MPXV infection) has historically been associated with more severe disease and higher case-fatality rates (CFR 5-11%). Common complications include secondary bacterial skin infection, pneumonia, bacteremia/sepsis syndromes, dehydration, obstructive lymphadenopathy and ocular manifestations.[1]

Ocular manifestations of mpox have been reported from the DRC since the 1970-80's, but have not been characterized systematically, documented in any detail, and are outdated.[2,3] These limited signals suggest common involvement of the eyelid/conjunctiva (9-23%); conjunctival involvement has been associated with more severe systemic disease.[4] Less common but potentially vision-threatening involvement of the sclera and the cornea (scleritis, keratitis) is also reported, as well as acute visual impairment and permanent blindness after corneal scarring.[3,5,6] In a concerning recent field survey in the mpox-affected province of Maniema in Eastern DRC, we found that up to 20% of former mpox patients report ocular sequelae, including blindness (unpublished results). Recent experience with clade II mpox suggests MPXV-related ophthalmic disease is primarily a disease of the ocular surface and the anterior eye. Uveitis has been reported, but very rarely during the recent outbreak. However, careful ophthalmic examination that includes the anterior chamber, the posterior chamber, the orbit and ocular adnexa has never been systematically conducted in mpox patients in DRC. As the burden of mpox increases in DRC, systematic, higher-resolution descriptive characterization of the nature and evolution of ocular complications in mpox patients is needed in order to understand, treat, and prevent vision loss or related morbidity.

PALM007 clinical trial

Currently, a randomized clinical trial of the safety and efficacy of the antiviral tecovirimat for mpox is ongoing the DRC, conducted by the Congolese National Institute for Biomedical Research (INRB), together with the National Institute of Allergy and Infectious Diseases (NIAID) and other partners (PALM007 trial, clinicaltrials.gov NCT05559099). The trial is screening and enrolling patients with mpox in two DRC sites (Tunda, Maniema Province; Kole, Sankuru Province).

Suspected mpox patients presenting in the involved regions are being referred to the PALM007 study site for screening and diagnosis, then are invited to participate in the clinical trial if mpox infection is PCR-confirmed. Trial participation involves randomization to treatment with either tecovirimat or a non-active placebo. Patients are hospitalized for at least 14 days and followed up daily until lesion resolution and negative blood PCR. After discharge, they are invited for follow-up on days 29 and 59.

MBOTE-Kamituga study

In late 2023, an outbreak of a new subclade MPXV Ib started in Kamituga health zone, a densely populated mining region in South Kivu. The region had not reported any cases of

mpox in recent decades until, in October 2023, the disease was first detected and began to primarily affect adolescents and young adults. So far, about 500 cases have been reported to health authorities, although the actual number of cases may be much higher due to significant underreporting. The outbreak is expanding into the surrounding regions, and has reached at least 10 DRC health zones by July 2024. Preliminary data shows that the epidemic is mainly due to heterosexual transmission and that up to 30% of cases are reported by sex workers, many of whom come from Burundi, Rwanda, Uganda and Tanzania. Since June 2024, our team is performing an observational study, called Mbote-Kamituga at Kamituga General Hospital which has a study schedule similar to the PALM007 study, with a baseline evaluation upon diagnosis and subsequent follow-up during hospitalization, and after discharge on days 29 and 59. Several cases have been reported with severe ocular lesions, resulting in blindness in some.

5 Study Objectives and Outcomes

Primary objective	
To determine the prevalence of **eye disease and visual deficiencies** at presentation	Composite presence or absence of:<br>• orbital lesions<br>• affection of the adnexa<br>• affection of the anterior segment<br>• affection of the posterior segment<br>• affection of the optic nerve
**Secondary objectives**	
To describe **all ocular symptoms, and signs** at presentation and during follow-up	Presence or absence of individual symptoms and signs over time
To assess **visual acuity** at presentation and during follow-up	Result of Optotype letters/numbers and/or E test over time
To determine the **presence of MPXV in tear fluid** at presentation and during follow-up	Result of MPXV-PCR on conjunctival swabs over time

6 Study Design

This is a prospective observational cohort study.

7 Methods

7.1 Study Setting, Population and Sampling Strategy

Mpox-patients will be recruited among the patients attending one of the clinics where the PALM007 trial is currently ongoing (Tunda General Hospital, Maniema Province and Kole General Hospital, Sankuru province), or where MBOTE-Kamituga is being conducted (Kamituga General Hospital, South-Kivu province). Recruitment in the present study may be continued after interruption or cessation of the PALM007 trial and the MBOTE study, and may be extended to new sites in the DRC where mpox cases are managed.

Mpox patients will be asked to participate in the MPOX-EYE study as soon as possible after their mpox diagnosis. They do not have to be enrolled in the PALM007 trial or MBOTE-Kamituga to be eligible for MPOX-EYE.

7.1.1 Sample Size and Power

The primary endpoint is the prevalence & description of ocular problems of mpox patients at presentation. Sample size should be as high as possible, depending on the evolution of the epidemic. A sample size of 62 patients would allow to detect a prevalence of 20% of acute

complications with a confidence level of 95% and a margin of error of 10%. Recruiting 246 participants allows to detect the same prevalence with a margin of error of 5%.

7.1.2 Inclusion Criteria

- Patient of any age or sex with laboratory-confirmed infection with MPXV
- Laboratory confirmation of MPXV infection less than 48 hours before inclusion
- Patient or culturally acceptable representative is willing and able to give informed consent or assent for participation in the study

7.1.3 Community participation

The local health authorities, surveillance team and hospital health care workers are informed about the purpose of the project and involved at an early phase in the design of the study. These local stakeholders provided valuable input in sensitive matters such as incentives for participation.

The study will be started in close collaboration with the PALM007 study, which has a dedicated social mobilization team.

Lastly, the study is done in collaboration with the national mpox program of the DRC Ministry of Health.

7.2 Procedures

7.2.1 General overview of study visits

All study visits will take place at a study hospital, taking into account the infection-prevention measures appropriate for the situation.

The following visits will be done:

- At diagnosis of mpox
- At day 14 post inclusion (+/- 3 days), only for hospitalized patients
- At day 29 post inclusion (+/- 7 days)
- At day 59 post inclusion (+/- 7 days)
- During unscheduled visits between enrolment and the end-of-study visit (day 59) upon presentation of new or worsening ocular symptoms based on a 3-point checklist.

7.2.2 Procedures per visit

Eligible participants will be informed about the study by their treating physician and referred to the study team if they are interested in participating.

A. *Screening and baseline visit*

- A member of the study team will inform the candidate about the study related procedures as part of the informed consent procedure. They will be invited to sign an informed consent or assent form (ICF). Once signed, participants can undergo the following study procedures
- Filling in a questionnaire concerning demographics, relevant medical history, concomitant medication, and past and current eye symptoms
- Systematic clinical and technical ophthalmological examination, by an ophthalmologist or other trained eye care provider who is a member of the study team (see below*)

B. Unscheduled visit between enrolment and end-of-study-visit

- Unscheduled visits are triggered by:
  - new or worsening reduced visual acuity
  - new or worsening photophobia
  - new or worsening significant eye pain
  - new or worsening exterior eye-lid lesion or edema
- Hospitalized patients will be asked on a daily basis for these symptoms by an attending health care worker
- Non-hospitalized patients will be encouraged to present themselves for an unscheduled visit if any of the three above-mentioned problems occur or worsen.
- Study activities during unscheduled visits:
  - Filling in a questionnaire concerning intercurrent and current eye symptoms and concomitant medications
  - Systematic clinical and technical ophthalmological examination, by an ophthalmologist or other trained eye care provider who is a member of the study team (see below*)

C. Follow-up visits at day 14 (+/- 3 days), day 29 (+/- 7 days) and day 59 (+/-7 days)

- Filling in a questionnaire concerning intercurrent and current eye symptoms and concomitant medications
- Systematic clinical and technical ophthalmological examination, by an ophthalmologist or other trained eye care provider who is a member of the study team (see below*)

* Systematic clinical and technical ophthalmological examination, by a study ophthalmologist or other trained eye care provider , including:

- Evaluation of visual acuity (optotype test)
- Evaluation of pupillary light reflex and eye movements
- Examination and photographic imaging of the anterior segment using slit lamp examination before and after application of fluoresceine
- Evaluation of quantitative (Schirmer test) and qualitative (Break up time) tear secretions

Individual aspects of this extensive assessment can be modified or omitted if the participant is not willing or capable of undergoing them (e.g. in young children) and will not be considered a protocol deviation.

The use of smartphone extensions for photographic imaging of the anterior and posterior eye segments enables a practicable but thorough ophthalmological evaluation without the need for close face-to-face contact. An ophthalmologist can then interpret the results of these tools after the study visit to determine the signs of disease present in the patient (cfr study outcomes).

7.2.3 End of study

Participation in the study ends after the end-of-study visit on day 59.

Participants that withdraw for other reasons are considered lost to follow-up.

	Baseline, at mpox diagnosis	Daily in case of hospitalization	Day 14 (+/- 3), only for hospitalized patients	Day 29 (+/- 7)	Day 59 (+/- 7)	Unscheduled
	**Study visit**					
**Screening and recruitment**						
Eligibility check	X					
ICF	X					
Demographics, concomitant medications and medical history	X					
**Ophthalmological assessment**						
Checklist for alarm signs		X				
Questionnaire	X		X	X	X	X
Evaluation of visual acuity (optotype test)	X		X	X	X	X
Evaluation of pupillary light reflex and eye movements	X		X	X	X	X
Examination and photographic imaging of the anterior segment using slit lamp examination before and after application of fluoresceine	X		X	X	X	X
Evaluation of quantitative (Schirmer test) and qualitative (Break up time) tear secretions	X		X	X	X	X
Dilated fundoscopic exam and photographic imaging of the retina	X		X	X	X	X
**Laboratory sampling**						
Conjunctival tear swab	X		X	X	X	

7.2.4 Laboratory Procedures

A. General comments

Tear fluid samples (conjunctival swabs) will undergo qPCR for MPXV. Since the results of this test will have no impact on clinical care, study samples will be analysed in batch at a later time point. The test results will therefore not be communicated to participants or the treating physicians.

The study will ensure that all laboratory activities, including transportation, processing, analysis, reporting and storage of samples, will be conducted in accordance with the GCLP. The laboratory will perform diagnostic testing in accordance with standard operating procedures and EN-ISO 15189. A laboratory analytical plan will be created prior to the start of the study describing all aspects in accordance with the WHO GCLP and specific Standard Operating Procedures (SOPs) containing additional details will be developed.

B. Biosafety

Measures are taken to minimize the risk of transmission when examining and sampling study participants and when manipulating clinical specimens from mpox patients, such as limiting the number of authorized staff, wearing appropriate personal protective equipment (PPE), using rigorously applied standard precautions, and avoiding any procedures that could generate infectious aerosols.

Study staff has normally been vaccinated with Jynneos®, a modified vaccinia Ankara, a replication-deficient smallpox vaccine as part of the PALM008 trial.

Manipulation and testing of clinical specimens will be carried out in a Biosaftey Level-3 (BSL-3) laboratory facility until effective inactivation of the sample. All specimen manipulations will be carried out with the necessary safety precautions by qualified staff wearing appropriate PPE.

Any personnel exposed to mpox patients or MPXV contaminated samples or materials without proper PPE, will be followed-up for 21 days after exposure for symptom surveillance regardless of vaccination status.

C. Sampling Procedures

Participants will be sampled by health care professionals equipped with the proper personal protective equipment: gloves, gown, FFP2 mask and face shield.

Tear fluid sampling will be performed as in standard clinical practice and outlined in the SOP, swabbing the palpebral conjunctiva from inner to outer canthus, avoiding the skin of the eyelids and the cornea with a flocked nylon swab.

D. PCR testing

MPXV PCR testing will be performed on site or in a central INRB reference laboratory through the RADI mpox detection kit following the instruction of the manufacturer (www.khmedical.co.kr) or another real-time PCR platform validated by the INRB.

E. On site storage and transport to the central lab

All samples will be stored at -20 or -60 freezers on-site for a short-term storage and then transferred to the INRB Goma or Kinshasa Biorepository for long-term storage.

7.2.5 Biobanking

After the initial analysis, left-over samples will be stored at -80°C and registered centrally at the INRB biobank in Kinshasa for a maximum of 20 years. Sample storage will be discussed during the informed consent procedure and subjects will have to indicate in the informed consent form (ICF), whether or not they agree with long-term storage. Stored samples will be used to further explore determinants and biomarkers of the disease and immune response, based on the primary analyses of this study or future scientific insights. Such analyses could be performed on the whole sample set or in a nested case-control design. The decision on whether the specimens can be used for future research will be taken by the investigators of the study and only after receiving approval from the Institutional

Review Board (IRB) and relevant Ethical Committee, including the Democratic Republic of the Congo's national ethics committee.

7.3 Data Analysis

7.3.1 Quantitative Data Analysis

The number of participants screened and enrolled or excluded will be summarized according to reason for exclusion. Of the enrollees, the number of patients discontinued or lost to follow-up will be recorded by reason and time of discontinuation. These figures will be summarized in a CONSORT flow diagram.

The prevalence of ocular manifestations will be described as the proportion of individuals presenting with the outcome with a 95% confidence interval.

8 Ethical Issues

8.1 Ethical (and regulatory) Review

This study will be submitted for formal review and approval to the following Institutional Review Boards (IRB):

A. Democratic Republic of the Congo's national ethics committee
B. The Institutional Review Board of ITM, Belgium
C. The Ethics committee of the Antwerp University Hospital (UZA), Belgium

The study will be carried out according to the principles stated in the Declaration of Helsinki, and any further update, the Good Clinical Practice of the WHO, and all other applicable national and international regulations, and according to established international scientific standards. The study will be prospectively registered in the publicly accessible clinical trial register www.clinicaltrials.gov.

8.2 Obtaining Informed Consent

Potential participants will be asked to provide informed consent before undergoing any study-specific procedures.

The Informed Consent Forms (ICF) as well as the informed consent procedure will describe the purpose of the study, the procedures to be followed, the risks and benefits of participation and the voluntary nature of participation. Study participants will be informed that participation in the study is completely voluntary, that the refusal to participate has no impact on their medical treatment, and that the participant can withdraw from the study at any time without any negative consequences or any implications on routine care. Mpox patients who decide not to participate in this study will have the same rights as participants to receive ophthalmological care, until day 59 after onset of mpox symptoms.

The informed consent (IC) procedure will be conducted in the native or preferred language of the patients by a qualified person formally identified by the principal investigator (PI). Written information and consent forms will be provided to the patients for their review in French and Swahili (Tunda) and Tetela (Kole). The written information and consent form will be translated in Swahili and Tetela by a native speaker. The translated ICFs will be submitted to national review boards for approval.

Patients will be given enough time to ask questions and to take a free decision. If the individual is willing to participate, he/she will be given the ICF and the content will be read by the participant or, if illiterate, by an independent witness to the participant. If the participant freely confirms the willingness to participate, he/she will be invited to confirm this by signing the ICF, adding his/her name (or thumb-printing if illiterate). The form will

be countersigned by the recruiting study staff. The participant will receive a completed copy of the ICF to keep. If a participant (or guardian) is unable to read or write, an independent witness must be present during the informed consent interview. The signature of the independent witness will be obtained in addition to the participants' thumbprint.

8.3 Assent Process

Parental/guardian permission and minor assent for children through 17 years of age will be obtained according to local standards and country-specific requirements. In DRC an assent must be obtained for children aged 12 to 17 years. Children that are too young to sign an assent form can be enrolled provided informed consent is signed/marked by a parent or guardian. If a minor participant expresses reluctance to participate in the study during the assent process, he or she will not be enrolled, notwithstanding consent from his or her parents/guardians.

8.4 Risks and Benefits of Participation

The risks for individuals of participating in the study are minimal. The four most important risks concern 1/ risks associated with biological sampling, 2/ risks associated with the administration of mydriatics and fluorescein during ophthalmological examination, 3/ loss of data confidentiality, 4/ loss of time for the participant.

1) The risks and inconveniences caused by conjunctival swabbing are minimal.
2) Patients with a medical contra-indication for the administration of mydriatics (e.g. presence of hyphema, patients using miotic therapy for glaucoma, who have a risk or history of iridocorneal angle closure and patients who need to drive,) or fluorescein or its constituents (e.g. prior history of hypersensitivity to fluorescein [note: anaphylactic reactions to topically administered fluorescein are extremely rare]) will be excluded from undergoing the procedures in which administration of the respective product(s) are involved.
3) Risks of loss of data confidentiality and privacy are reduced as much as possible (see section 12.3 on data confidentiality). Participants may be asked questions about private matters such as their medical history and age, which may be uncomfortable or embarrassing. Participants may refuse to answer any question they do not feel comfortable disclosing.
4) Considering time lost by participants: study activities will either be performed during hospitalization, planned in combination with follow-up visits for the PALM007 or MBOTE-Kamituga study, or planned separately, together with the participants to minimize the loss of productive time. Participants will be compensated for the study visits at days 29 and 59 if they are scheduled outside of hospitalization. In this case the participant and up to two individuals accompanying them will receive US$15, equivalent of 30,000 Congolese francs in local currency plus reimbursement of round-trip travel to the study site. For unscheduled study visits in case of new or worsening eye symptoms after discharge, re-imbursement of travel will be provided for the participant and up to two individuals accompanying the participant. Compensation for the current study cannot be cumulated with compensation received for visits attended in the framework of another study such as PALM007 and MBOTE-Kamituga.

Participation in the study offers both direct and indirect benefits to patients. Participation in the study is free of charge and participants will benefit from detailed eye examination and evaluation by a dedicated ophthalmologist or other trained eye care provider, and immediate (supportive) care for potential eye manifestations, which is expected to reduce the risk of complications.

Care for ophthalmological problems is provided free of charge by the study to mpox patients irrespective of inclusion in this study, starting from mpox diagnosis until the end-of-study visit (day 59) and consists of the following:

- Anti-inflammatory eye drops: corticosteroids, NSAIDs, antihistaminics
- Mydriatic eye drops: anticholinergic cycloplegic for therapeutic purposes, tropicamide for diagnostic purposes
- Pain killers/anaesthetic eye drops: Tetracaine ou oxybuprocaine
- Artificial tears: Methylcellulose, Sodium chloride
- Antibacterial eye drops/ointments: quinolones, aminoglycosides, tetracyclines
- Antiviral eye drops: trifluridine

In the event that eye conditions require a treatment not present in this list or examinations not described under the study procedures in section 7.2.2, the patient will be informed of the diagnosis and necessity of additional treatment or examination, but the study will not be able to provide this treatment or examinations nor cover the costs for referral and treatment of such eye conditions.

Information gleaned from this research study will increase our understanding of mpox. This information will help to decrease the burden of mpox in the affected individuals and populations.

8.5 Vulnerable populations

8.5.1 Impoverished people

The target populations are located in a heavily impoverished region and patients often refrain from seeking medical care due to financial constraints. A careful balance is, therefore, at play. While participants will receive conservative treatment for acute mpox-related eye conditions and compensation for their time loss, we will also ensure that the anticipated remuneration for study involvement does not serve as a primary motivation for participation.

8.5.2 Children

Children are most affected by mpox. Information on the disease in this age group is therefore crucial. As this is an observational study, the risk of participation is minimal.

8.5.3 Pregnant and lactating women

As this protocol foresees only limited biological sampling and no other intervention, there is no specific risk for pregnant women, and they can safely be included.

8.6 Insurance

The Sponsor of this study, ITM, has obtained a (no-fault) study insurance to cover any injury, damage or loss to study participants which is caused directly or indirectly by their participation in the study. Participants who suffer injury from study-related procedures will be entitled to free medical treatment for such injury, and to such financial assistance as would compensate them equitably for any resultant impairment, disability or handicap, as explicated in the ICF.

8.7 Confidentiality and privacy

The study investigators will be responsible for capturing and storing the participants' personal details and study identification code in a subject identification code list. The personal details of participants, including names and contact details, will be noted in a separate register that is only accessible to the local study team. This register will enable proper follow-up. To ensure confidentiality, this list will be kept in a separate, locked cupboard together with the signed ICFs and only authorized members of the study team will

have access to it. All participant information will be stored in locked filing cabinets in areas with access limited to study site staff.

At inclusion, each participant will be allocated a study ID number, ensuring pseudo-anonymization of the participants. All participant's information and laboratory specimens, including stored specimens, reports, study data collection, process, and administrative forms will be identified by the study ID, date of data or sample collection and study visit. Names will not be used and all study researchers will only have access to the pseudonymized data in the database.

Images will be captured with a dedicated password protected study smartphone and saved into a secure location. Only close-up images of the anterior chamber and the retina will be taken, virtually eliminating the risk for identification.

The database will be secured with password-protected access systems. Participant's study information will not be released to anybody outside the medical team, except as necessary (and under confidentiality agreement) for independent monitoring, auditing and for inspection by competent authorities.

During or after final publication of results, individual patient data might be publicly shared on an anonymized basis, in line with ITM (open access) data sharing policy.

9 Monitoring And Quality Control

Detailed laboratory Standard Operating Procedures (SOPs) will be developed to cover all laboratory processes from sample collection up to shipment, assays and storage. All tests will be performed by qualified laboratory technologists. The PIs from INRB will take responsibility to ensure that all study specific analyses will be conducted in accordance to GC(L)P guidelines, study specific SOPs, and for internal/external quality control systems of the respective assays. During study visits, protocol and GC(L)P compliance will be monitored, including proper data management and documentation.

10 Timelines

- Submission EC: July 31, 2024
- Study start: September 2024
- Duration: 1 year, to be extended

11 Collaborative partnership

The study will be led by INRB in close collaboration with ITM and the following partner in the field:

- **National Institute of Allergy and Infectious Diseases (NIAID) and Leidos Biomedical Research, inc**. are leading partners in the PALM007 study. Collaboration with these insitutes will ensure coordination and synergy between the two different projects. They provided input into the protocol and will be involded in study conduct and data analyse. In addition, they will support with logistics of the study throught the PALM007 study. Key persons involved are:
    - Dr. Olivier Tshiani Mbaya
    - Dr. Lori Dodd
    - Dr. Veronique Nussenblatt
    - Dr. Ian Crozier

12 Data Management And Archiving

12.1 Data Management Responsibilities

Although the Principal Investigator has ultimate responsibility for data management, practical implementation and monitoring will be carried out by the INRB Data Manager in collaboration with study staff at the sites. This will be done in accordance with the study's data management plan (DMP), which broadly describes the aspects below. The ITM Data Manager and other ITM colleagues will help support the implementation of this DMP.

12.2 Data Collection Methods

Study data will be collected on paper and/or directly via electronic questionnaires in the study database using REDCap. Only data defined by the study protocol will be collected.

REDCap (Research Electronic Data Capture) is a software program widely used in the academic research community to create and manage surveys, databases and research studies. It is a secure web application as well as a mobile application that can be used to collect, cleanse, and manage subject information in compliance with various applicable standards and regulations (GCP, CFR 21 part 11, FISMA, HIPAA). Its mobile application enables offline data collection with smartphones or tablets. In addition, the software includes, among other things, a query management system and capabilities to import and export data in various forms.

Paper questionnaires will be used where necessary. Edit controls and branching logic, programmed on the electronic forms, will validate at the point of data entry and support data quality. Images will be captured directly in REDCap. To ensure better data quality, appropriate data management training will be conducted before the actual data collection (and processing) begins. In addition, the data will be reviewed and monitored during the study by various study collaborators to obtain a timely database lock.

For MPOX EYE study participants who also participate in the PALM007 trial and MBOTE Kamituga study, information relevant to the MPOX EYE study that has already been obtained during assessments for those studies may also be extracted from the participants' source documents rather than asking the participants the same questions twice. This may also eliminate inconsistent data between studies.

12.3 Data Security And Confidentiality

The computer/data management system to be used includes a robust security architecture including encryption, firewalls, anti-virus software, and controlled user access (tablet/computer authentication, REDCap username, personal password, and authorized user role). Data backup will be provided at the server, computer, mobile device, and/or database level, if applicable.

ITM and INRB will also ensure that the necessary steps are taken to ensure the retention of all data management documents (Data Management Plan, procedures, completed questionnaires) and that IT equipment (tablets, computers, server) will be secured (locked closets, locked offices and/or rooms and/or controlled by badge).

Study participants' private data will be treated as confidential. Names of study participants and contact data will be replaced by a specific identification code of the study subject (= pseudonym). Only pseudonymized data will be used and distributed within the larger study team. Pseudonymized images will be captured and stored in REDCap. Pseudonymized

pictures can be shared within the study team for remote ophthalmological evaluation and guidance on patient management. Images can be stored in the dedicated password protected study smartphone for the duration of the study. After synchronisation with the online study database, these local copies will be erased.

12.4 Data Sharing

The study will make its research data available for secondary research and to support both clinical care and the Congolese surveillance and response to mpox, considering the following key aspects:

- ITM's data sharing policy will be followed, with the following key aspects mentioned below.
- European FAIR data principles ("Findable"; "Accessible"; "Interoperable"; "Reusable") will also be followed ("as open as possible, as closed as necessary")
- Ensuring confidentiality by anonymizing its data (among other things, by replacing the study subject's identification code with a new code that can no longer be used to identify the patient; generalizing and randomizing specific variables).
- Ensuring that ethical approvals have been obtained by ethics committees and by study participants.
- Provision of data, primarily medical, personal, and sensitive, through a governed data access mechanism and along a transparent decision-making process, with:
    i. Completion of a data request form,
    ii. Evaluation through a data access committee,
    iii. agreement to share data by that committee,
    iv. secure transfer of anonymized data,
    v. additional metadata that clarifies the research data. The metadata will include the study protocol, questionnaire(s), data dictionary.

12.5 Archiving

The principal investigator is responsible for the retention of all essential documents listed in the Good Clinical Practice guideline: Informed Consent (Form) or IC(F) or Informed Consent Form. All essential documentation for all study subjects must be retained by the principal investigator in a secure storage facility for a minimum of 20 years in accordance with INRB policies or local country regulatory requirements (whichever is longer). The DRC requires that study records be retained for up to 2 years after publication. These records must also be retained in accordance with the ethics committee's medical record retention requirements, whichever is longer. All stored records must be kept confidential to the extent required by local, state, and national laws of the jurisdiction in which they are stored.

13 Dissemination of Results

The results will be made available and discussed with the health facilities, the Provincial Health Bureau and the Ministry of Health at all levels. Based on the result we will be advocating for the revision of mpox management guidelines.

The study will be submitted to a peer reviewed open access journal for publication and presentation at national and international conferences.

14 List Of Abbreviations

DMP	Data Management Plan
DNA	Deoxyribonucleic acid
GCP	Good Clinical Practice
GDPR	General Data Protection Regulation
GLP	Good Laboratory Practice
IC(F)	Informed Consent (Form)
(I)EC	(Independent) Ethics Committee
INRB	Institut National pour la Recherche Biomédical
IRB	Institutional Review Board
ITM	Institute of Tropical Medicine
MPXV	Mpox virus
PCR	Polymerase chain reaction
PI	Principal Investigator
QC	Quality Control
qPCR	Quantitative PCR
RCT	Randomized controlled trial
SDV	Source Data Verification
SOP	Standard Operating Procedure

15 Annexes

- Informed Consent Form(s)

16 References

1. Van Dijck, C. *et al.* Emergence of mpox in the post-smallpox era - a narrative review on mpox epidemiology. *Clin. Microbiol. Infect.* (2023). doi:10.1016/j.cmi.2023.08.008

2. Breman, J. G., Ruti, K. & Steniowski, M. V. Human monkeypox, 1970-79. *Bull. World*

Health Organ. **58**, 165–182 (1980).

3. Jezek, Z., Grab, B., Szczeniowski, M., Paluku, K. M. & Mutombo, M. Clinico-epidemiological features of monkeypox patients with an animal or human source of infection. *Bull. World Health Organ.* **66**, 459–464 (1988).

4. Pittman, P. R. *et al.* Clinical characterization and placental pathology of mpox infection in hospitalized patients in the Democratic Republic of the Congo. *PLoS Negl. Trop. Dis.* **17**, 1–28 (2023).

5. Ježek, Z., Szczeniowski, M., Paluku, K. M. & Mutombo, M. Human Monkeypox: Clinical Features of 282 Patients. *J. Infect. Dis.* **156**, 293–298 (1987).

6. Osadebe, L. *et al.* Enhancing case definitions for surveillance of human monkeypox in the Democratic Republic of Congo. *PLoS Negl. Trop. Dis.* **11**, 1–13 (2017).
