# Supplementary material for: Prevalence, characteristics, and clinical course of mpox-related ophthalmic disease (MBOTE-EYE): a prospective cohort study in South Kivu, Democratic Republic of the Congo
Source: Lancet Glob Health. Author manuscript; Available in PMC 2026 Oct 9. (PMC13646179; doi:10.1016/j.langlo.2026.104054)
Supplement: Supplementary file 5 [file NIHMS2208071-supplement-5.pdf]

THE LANCET
Global Health

Supplementary appendix 5

This appendix formed part of the original submission and has been peer reviewed.
We post it as supplied by the authors.

Supplement to: Kabesha T, Van Dijck C, Mudwanga S, et al. Prevalence, characteristics, and clinical course of mpox-related ophthalmic disease (MBOTE-EYE): a prospective cohort study in South Kivu, Democratic Republic of the Congo. *Lancet Glob Health* 2026. https://doi.org/10.1016/j.langlo.2026.104054

Equitable Partnership Declaration

If any questions do not apply to your study, please indicate "N/A" for "not applicable.
For more information on how to complete this form see the Information for Authors document.

Researcher considerations

1. Please detail the involvement that researchers who are based in the country or countries of study had during a) study design; b) clinical study processes, such as processing blood samples, prescribing medication, or patient recruitment; c) data interpretation; and d) manuscript preparation, commenting on all aspects. If they were not involved in any of these aspects, please explain why.

   This should include a thorough description of their leadership roles in the study. Are local researchers named in the author list or the acknowledgements, or are they not mentioned at all (and, if not, why)? Please also describe the involvement of early career researchers based in the location of the study. Some of this information might be repeated from the Contributors section in the manuscript. Note: we adhere to [ICMJE authorship criteria] for naming authors on a paper.

**a) Study design:**<br>The study was co-designed by researchers based in the Democratic Republic of the Congo, including Theophile Kabesha (*early career researcher)*, Samuel Mudwanga (*early career researcher*), Olivier Tshiani-Mbaya, Sabin Sabiti Nundu, and Placide Mbala-Kingebeni, alongside international collaborators. These local investigators contributed to conceptualisation and protocol development, ensuring that the study design was contextually appropriate and aligned with local clinical realities and infrastructure.
**b) Clinical study processes:**<br>Researchers based in the DRC were centrally involved in all clinical study processes. Theophile Kabesha, Samuel Mudwanga, Guy Mukari (*early career researcher)*, and Papy Munganga (*early career researcher)* led participant inclusion, clinical investigation, sample and data collection on the ground. Additional local collaborators (including those affiliated with INRB, Université Officielle de Bukavu) contributed to laboratory procedures, sample handling, and implementation of study protocols.
**c) Data interpretation:**<br>Local researchers, including Theophile Kabesha, Samuel Mudwanga, Yves Mujula (*early career researcher*), Sabin Sabiti Nundu, and Placide Mbala-Kingebeni, contributed to data verification and interpretation. They provided critical contextual insight into the findings, ensuring that analyses were interpreted in light of local epidemiology, healthcare structures, and patient characteristics.
**d) Manuscript preparation:**<br>Researchers based in the DRC were actively involved in manuscript preparation at all stages. Theophile Kabesha and Samuel Mudwanga drafted the initial manuscript, while multiple local authors participated in manuscript revision and critical review. All authors, including those based in the DRC, reviewed and approved the final version. Local investigators also contributed to decisions regarding submission, ensuring equitable ownership of the research output.

2. How was funding used to remunerate and enhance the skills of researchers in the countries of study? And how was funding used to improve research infrastructure at the study sites?

   Potentially effective investments into long-term skills and opportunities within local institutions could include training or mentorship in analytical techniques and manuscript writing, opportunities to lead all or specific aspects of the study, financial remuneration rather than requiring volunteers, and other professional development and educational opportunities.

   Improvements to research infrastructure could include funding extended trial designs (eg, platform trials), establishment of long-term contracts for research staff, building research facilities, and setting up local control of funding allocation.

**Skills:**<br>Researchers from INRB, Kinshasa (DRC) together with researchers from ITM, Antwerp (Belgium) provided extensive training on study procedures, good clinical practice, and good laboratory practice to the field study team in Kamituga (DRC), and to interested students from Université Officielle de Bukavu. This activity was supported by funding from ALERRT and the Belgian Directorate-General for Development Cooperation and Humanitarian Aid, and the Department of Economy, Science, and Innovation of the Flemish Government.<br>The PCR analyses at INRB lab were performed by INRB researchers and lab technicians in the context of a long-term partnership and capacity sharing infrastructure between ITM and INRB funded by ALERRT and DGD.<br>Theophile Kabesha (based in Bukavu, DRC) is doing for a fully funded 12-week on-site research course at ITM Antwerp (Belgium).
**Research infrastructure:**<br>The study's main budget (funded by IMReC grant MRR-184813) was entirely managed by INRB, Kinshasa (DRC). The budget was used to buy consumables, lab reagents, to pay study staff, and to procure ophthalmological equipment that will be used during subsequent studies under the lead of INRB.<br>Laboratory infrastructure for mpox diagnostics and research at Kamituga General Hospital was funded by EDCTP3 and the Belgian Directorate-General for Development Cooperation and Humanitarian Aid.

3. How did you safeguard the researchers who implemented the study?

   Please describe how you guaranteed safe working conditions for study staff, including provision of appropriate personal protective equipment, protection from violence, and prevention of overworking.

Safe working conditions for study staff were ensured through multiple complementary measures. All personnel involved in clinical and laboratory procedures were trained in infection prevention and control (IPC), including correct donning and doffing of personal protective equipment (PPE), in line with international and national guidelines. Appropriate PPE (e.g., gloves, gowns, masks, and eye protection) was provided.<br><br>Clinical activities involving potentially infectious patients were conducted in designated areas following established biosafety protocols. Laboratory procedures were performed in dedicated facilities (the mpox laboratory at Kamituga General Hospital, and the central laboratory at INRB Kinshasa) with trained personnel and adherence to biosafety standards.

To mitigate risks related to workload, study responsibilities were distributed across a multidisciplinary team, with clear task allocation and supervision. Work schedules were organised to avoid excessive working hours, and regular supervision ensured adherence to safety protocols and staff well-being.

Protection from potential security risks was addressed through collaboration with local health authorities and NGOs (primarily, ALIMA). This facilitated a controlled and secure working environment for staff.

Benefits to the communities and regions of study

4. How does the study address the research and policy priorities of its location?

   How were the local priorities determined and then used to inform the research question? Who decided which priorities to take forward? Which elements of the study address those priorities?

The study was explicitly aligned with national and local research and public health priorities in the DRC.
Local priorities were first identified by a consortium of Congolese institutions, including INRB (Kinshasa and Goma), the Université Officielle de Bukavu, the Université Catholic de Bukavu, and clinical partners (ALIMA).
These priorities were explored jointly by local and international investigators, with a leading role for DRC-based researchers (including Placide Mbala-Kingebeni, Theophile Kabesha, and collaborators at INRB and affiliated institutions), who contributed critical insight into the most pressing clinical and research gaps. Key priorities included improving clinical characterisation of disease manifestations, strengthening diagnostic and laboratory capacity, and generating data to inform patient management and public health responses.
The research question and study design were directly shaped by these priorities. Specifically, the study focused on systematic clinical data collection, laboratory confirmation, and detailed phenotyping of patients, which address gaps in evidence needed for clinical guidelines. The inclusion of a local clinical site and laboratories ensured that the research was embedded within existing healthcare and surveillance structures and contributed to capacity strengthening.
Furthermore, the involvement of national stakeholders in study design, implementation, data interpretation, and manuscript preparation ensured that the findings are relevant and readily translatable into policy and practice within the DRC.

5. How will research products be shared in the community of study?

   For instance, will you be providing written or oral layperson summaries for non-academic information sharing? Will study data be made available to institutions in the region(s) of study? The Lancet Group *encourages authors to translate the summary (abstract) into relevant languages after paper editing; do you intend to translate your summary?*

Research findings will be actively disseminated within the communities and institutions involved in the study through both academic and non-academic channels. Local dissemination will be led by Theophile Kabesha and collaborators from the Université

Officielle de Bukavu and Université Catholic de Bukavu, with a focus on accessible, community-oriented formats.

Planned activities include the organisation of conference-debates with students from local higher education institutions (including the Institut Supérieur des Techniques Médicales, Institut Supérieur de Développement Rural, and Institut Supérieur Pédagogique). In addition, community-facing seminars will be organized in local religious settings, including Protestant churches and Catholic parishes, to facilitate engagement with broader, non-academic audiences. These sessions will provide layperson summaries of the study findings and allow for dialogue with community members.
At the institutional level, study data and findings will be shared between Congolese partner institutions, including INRB and affiliated universities, supporting local ownership and further research use.
For wider dissemination, the authors intend to provide a lay summary of the study findings. In line with The Lancet Group recommendations, the abstract will be translated into French, Swahili, and Lingala.

6. How were individuals, communities, and environments protected from harm?

a) *How did you ensure that sensitive patient data were handled safely and respectfully? Was there any potential for stigma or discrimination against participants arising from any of the procedures or outcomes of the study?*

Sensitive patient data were handled in accordance with Good Clinical Practice (GCP) guidelines and applicable data protection regulations. Data were pseudonymised using unique study identifiers, and access was restricted to authorised study personnel only. Secure data management systems were used to store and process data, ensuring confidentiality and preventing unauthorised access.
The study team was aware that mpox may be associated with stigma and potential discrimination. To mitigate this risk, all efforts were made to protect participant confidentiality, including conducting clinical assessments in appropriate, settings, limiting the collection of identifiable information to what was strictly necessary, and ensuring that results were reported in an aggregated and anonymised manner.
In addition, study staff were used to handle interactions sensitively with participants and communities, emphasising respect, confidentiality, and non-discriminatory practices.

b) *Might any of the tests be experienced as invasive or culturally insensitive?*

Some study procedures, including blood sampling, conjunctival swabbing, and detailed ophthalmological examinations, may be perceived as invasive or uncomfortable by participants. To address this, all procedures were performed by trained healthcare professionals who were familiar with the local context and clinical practices. In addition, all study procedures were embedded in the standard clinical care provided to the study participants.
Prior to any procedure, participants were provided with clear explanations in their language, and informed consent was obtained. Efforts were made to ensure that procedures were conducted in a respectful and culturally sensitive manner, with attention to privacy and patient comfort.
No procedures were considered culturally inappropriate in the study context; however, continuous engagement with local staff and participants allowed the study team to remain responsive to any concerns and adapt practices if needed.

c) *How did you determine that work was sensitive to traditions, restrictions, and considerations of all cultural and religious groups in the study population?*

Cultural and religious sensitivity was ensured through the active involvement of local researchers, clinicians, and community-facing staff throughout all phases of the study. Investigators based in the Democratic Republic of the Congo, who are familiar with local customs, beliefs, and social norms, played a central role in designing and implementing study procedures.
Study materials, including informed consent forms and patient information, were adapted to the local context and delivered in appropriate languages. Continuous interaction with participants and communities during recruitment and follow-up allowed the study team to identify and address any concerns related to cultural or religious practices.
In addition, engagement with community structures, including local institutions and religious settings, provided further insight into community expectations and norms. This iterative, locally grounded approach ensured that study procedures remained respectful and acceptable across different cultural and religious groups within the study population.

d) *Were biowaste and radioactive waste disposed of in accordance with local laws?*

Biowaste produced at Kamituga General Hospital was disposed of in accordance with local laws, following procedures in place at Kamituga General Hospital, and with the support of the NGO ALIMA who is specialized in this matter.
Biowaste produced at INRB (Kinshasa and Goma) was disposed of in accordance with local laws, by the respective institutions.

e) *Were any structures built that would have impacted members of the community or the environment (such as handwashing facilities in a public space)? If so, how did you ensure that you had appropriate community buy-in?*

No such structures were built.

f) *How might the study have impacted existing health-care resources (such as staff workloads, use of equipment that is typically employed elsewhere, or reallocation of public funds)?*

The study did not place a significant burden on existing health-care resources. All study-related activities, including personnel, equipment, and consumables, were funded through dedicated study funds. This ensured that routine healthcare services were not disrupted, and no reallocation of public resources was required.
Study staff were specifically recruited or supported within the project framework, and any use of clinical or laboratory infrastructure was coordinated to avoid interference with standard patient care.

7. Confirm that local ethics review was sought, and please provide the approval number. If not sought, please explain why.

The study was approved by the Ethics Committee of the University of Kinshasa (approval IDs ESP/CE/78/2024 and ESP/CE/083/2024, respectively)

Secondary analyses

8. Have the data analysed in your study been extracted from another source, such as a national survey, rather than being directly collected by the authors of this paper?

No

If the authors of this paper were not involved in data collection, how were the findings interpreted with sufficient contextual knowledge?
The Lancet Group *believe contextual understanding is crucial for informed data analysis and interpretation.*

NA

---

9. Please provide the title (eg, Dr/Prof, Mr/Mrs/Ms/Mx), name, and email address of an author who can be contacted about this statement.

Name: Dr Laurens Liesenborghs
Email: lliesenborghs@itg.be

10. Finally, please provide the title and name of an author from one country of study who has seen and approved this form.

Name: Dr. Sabin Sabiti Nundu
